# Genetic structure and climate niche differentiation among populations of *Leopardus geoffroyi*

**DOI:** 10.1002/ece3.70223

**Published:** 2024-08-30

**Authors:** Alberto F. Fameli, Javier A. Pereira, Julio Rojo Gómez, María Jimena Gómez Fernández

**Affiliations:** ^1^ División Mastozoología, Museo Argentino de Ciencias Naturales “Bernardino Rivadavia” Consejo Nacional de Investigaciones Científicas y Técnicas (CONICET) Ciudad de Buenos Aires Argentina; ^2^ Department of Ecosystem Science and Management The Pennsylvania State University University Park Pennsylvania USA

**Keywords:** ecological niche modeling, Geoffroy's cat, *Leopardus geoffroyi*, microsatellites, niche differentiation, population genetic structure

## Abstract

Geoffroy's cat (*Leopardus geoffroyi*) is a small‐sized felid native to South America. Given the species' distribution covering a wide variety of habitats, and the presence of high levels of anthropization in part of its range, it is possible that genetically differentiated groups exist and that they occupy different climatic niches. We assessed patterns of contemporary genetic diversity and structure in the species across most of its range, characterizing each inferred genetic group based on ecological niche models. We genotyped 11 microsatellites for 142 samples covering most of Geoffroy's cat distribution, and investigated patterns of genetic structure and diversity, applying spatial and nonspatial Bayesian clustering methods and a spatial principal component analysis. We created ecological niche models for each genetic cluster, evaluating whether these clusters occupy different climatic spaces and display differences in the suitability of different values of the climatic variables analyzed. We identified two genetic clusters, one in the north‐northeast and the other in the south‐southwest of the species' distribution. These clusters showed moderate F_ST_ values between them and differences in dispersal/genetic diversity. We found isolation‐by‐distance patterns globally and within each cluster. We observed lower expected heterozygosity compared with other studies and a north–south gradient in allelic richness. The southern cluster showed lower genetic variability and a more restricted climatic niche suggesting that this group is more vulnerable to the effects of the current context of climate change. Individuals from the southern genetic cluster are under different pressures, likely a product of the particularly dry habitat they occupy. Climatic variables associated with habitat suitability suggest the southern cluster has affinity for the arid and semiarid conditions present in its distribution. Conservation measures should consider the genetic structure observed and differences in climatic spaces to maintain the evolutionary potential of the species.

## Graphical abstract

Geoffroy's cat (*Leopardus geoffroyi*) is a small‐sized felid present in a wide variety of habitats in South America, including areas with high levels of anthropization. We identified two genetic clusters, one in the north‐northeast of the species' distribution and the other in the south‐southwest, the latter showing lower genetic variability and a more restricted climatic niche, which suggests that this group is more vulnerable to the effects of the current context of climate change.

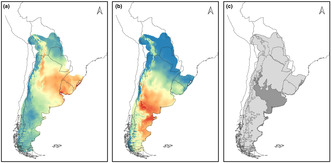

## 1 INTRODUCTION

**FIGURE 1:**
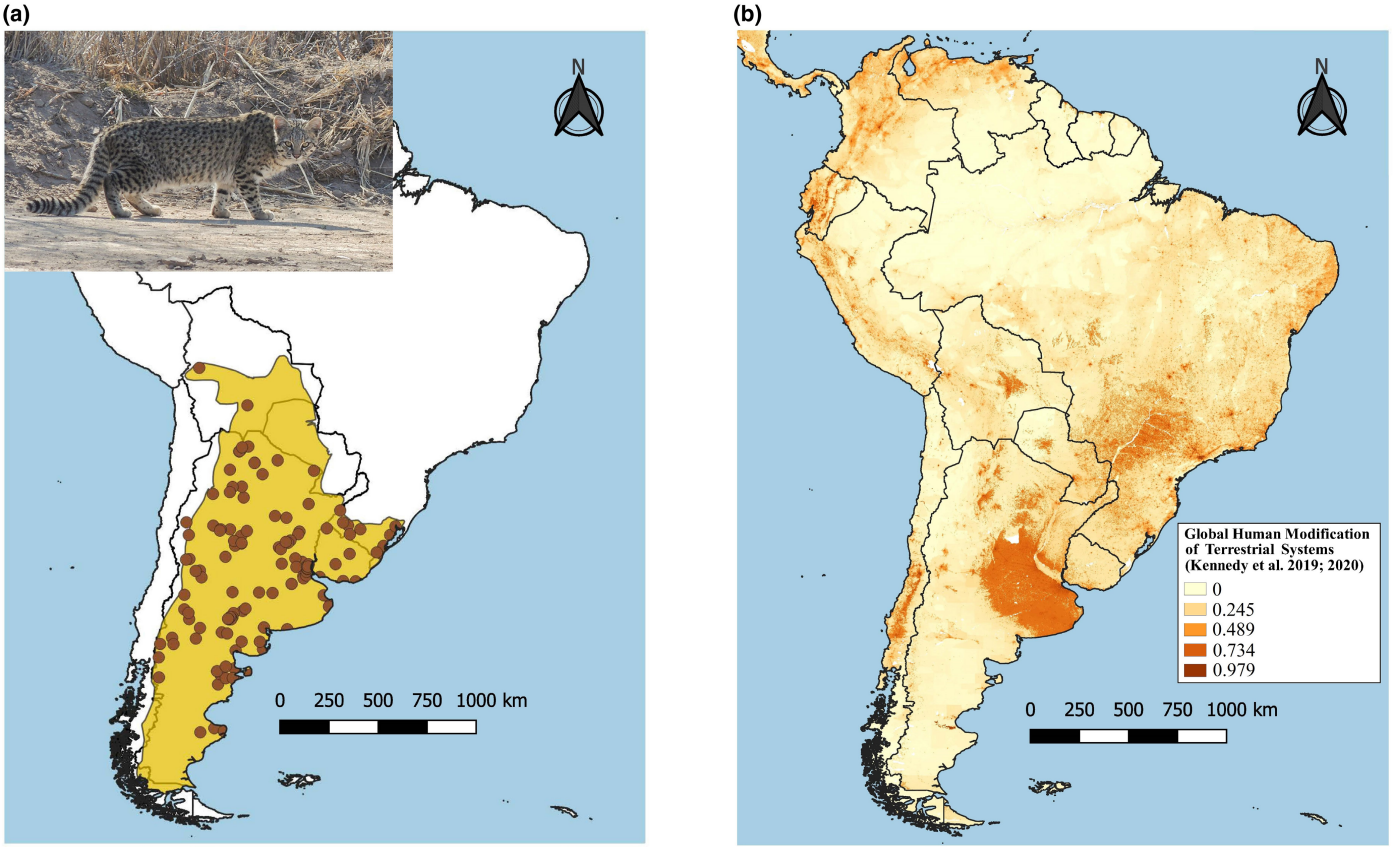
(a) Distribution of *Leopardus geoffroyi* (IUCN 2015). The circles indicate the geographic origin of samples used in this work. (b) Fragment of the Global Human Modification Map by Kennedy et al. (2019, 2020), a metric based on 13 anthropogenic stressors and their estimated impacts. Values closer to 1 represent higher anthropic modifications. Photograph: Adult Geoffroy's cat (*L. geoffroyi*), credit Lucía Martínez Retta.

Geoffroy's cat (*Leopardus geoffroyi*) is a small‐sized (4–5 kg) felid native to South America, with an extensive latitudinal range from Bolivia and Brazil to southern Patagonia in Argentina and Chile (Cuyckens et al., 2016; Pereira et al., 2015; Figure 1a). The species occurs in a wide variety of habitats including grasslands, temperate forests, deserts, shrublands, sub‐tropical forests, and marshes, from 0 to >3500 m.a.s.l. (Cuyckens et al., 2016; Ximénez, 1975). As a generalist and ecologically flexible carnivore, Geoffroy's cat is tolerant to habitat conversion due to human activities, being present in agricultural and suburban areas (Castillo et al., 2008; Guidobono et al., 2016; Pereira & Aprile, 2012). Remarkably, the species has relatively high dispersal ability, with documented long‐distance movements ranging from >25 km in southern Patagonia (Johnson & Franklin, 1991) to up to 128 km in central Argentina (Pereira & Novaro, 2014). Between the 1960s and late 1980s, this felid underwent heavy hunting pressure motivated by fur trade (Nowell & Jackson, 1996). Nearly 341,600 Geoffroy's cat pelts were exported from Argentina during the 1976–1979 period (Mares & Ojeda, 1984), resulting in the species becoming the most traded felid in the world by 1980 (MacMahan, 1986). Argentina prohibited domestic trade and reinforced export ban on native cats by 1986 (Broad, 1987). Currently, the species is categorized as “Least Concern” by the IUCN (Pereira et al., 2015). Habitat loss and fragmentation along with retaliatory killing due to poultry predation remain as the species' main threats, whereas potential negative impacts from current trends in climate change have also been proposed (Canepuccia et al., 2008; Pereira & Novaro, 2014).

Geoffroy's cat is a monotypic species, as recently supported through molecular (Gómez Fernández et al., 2020; Johnson et al., 1999), morphological and morphometric evidence (Nascimento, 2014). Mitochondrial DNA analysis indicated little effect of the last glaciation on the species' distribution pattern (Gómez Fernández et al., 2020); however, contemporary habitat changes might be affecting connectivity and distribution of genetic variability. The core of the species' distribution has undergone intensive transformation of natural habitats mostly due to agriculture, representing the highest level of anthropization in South America (Kennedy et al., 2019, 2020; Figure 1b). Despite the species' ability to move and survive in altered landscapes, it is unlikely that such a large‐scale habitat modification process has not reduced current connectivity among populations. As expected, a recent study of genetic connectivity of Geoffroy's cat across its range (Sartor et al., 2022) demonstrated that intensive agriculture imposes high resistance to movement due to absence of natural vegetation.

An assessment of contemporary population genetic variation of a species across its geographical range is crucial to understand conservation and management needs, including the identification of evolutionary significant units and management units (Coates et al., 2018; Crandall et al., 2000; Moritz, 1994). While patterns of genetic diversity and structure provide insights into the connectivity dynamics of a species, ecological niche models (ENMs) can characterize environmental factors associated with the geographical location of genetic structure. These ENMs usually assume that species are genetically uniform and have a single environmental niche that is stable in space and time (Guisan & Thuiller, 2005), which would be the equivalent to assuming that the species constitutes a single panmictic population throughout its entire geographical range (Gotelli & Stanton‐Geddes, 2015). However, in species with a wide geographical range that covers numerous biomes, such as Geoffroy's cat, diverse responses to environmental features can be expected. Therefore, complementing assessment of population genetic structure with niche modeling could show spatial restrictions to gene flow, which in turn could translate into the species occupying different ecological niches in heterogeneous landscapes (Gotelli & Stanton‐Geddes, 2015; Milanesi et al., 2018).

Our aim was to analyze the large‐scale patterns of nuclear genetic variability in Geoffroy's cat in southern South America, using samples representing most of the species' distribution range. Particularly, our objectives were (1) to assess the overall pattern of genetic structure and diversity using microsatellite loci and (2) to develop genetic ENMs grounded on climatic variables for each inferred genetic group. Because Geoffroy's cat displays a broad distribution, a heterogeneous array of environmental pressures is expected to occur across its range.

## 2 MATERIALS AND METHODS

### 2.1 Sample collection and lab procedures

We analyzed 142 samples covering most of Geoffroy's cat distribution (Figure 1a). Samples consisted of preserved tissues obtained from museum specimens, fresh muscle and blood samples collected from road‐killed and captured wild individuals, respectively, and feces collected in the field. Collection dates ranged from 1928 to 2015, and we divided samples into two groups: “historical” (period 1928–1990) and “contemporary” (period 1991–2015). Museum samples lacking clear information regarding origin were excluded. The year 1990 was used as the limit to separate samples before and after the export ban on native cats in Argentina (Broad, 1987).

We extracted DNA following Gómez Fernández et al. (2020) and amplified a panel of 11 microsatellite loci (F42, F53, F98, F124, F146, FCA391, FCA424, FCA441, FCA453, FCA723, and FCA742) developed for domestic cats (Menotti‐Raymond et al., 1999, 2005). PCR amplifications were performed individually using the M13‐tailed primer method (Boutin‐Ganache et al., 2001) to label amplicons with 5′‐fluorescent tags (6‐FAM, HEX or NED). Amplification conditions are provided in Appendix S1. We used a MegaBACE 1000 automated sequencer (GE Healthcare) and an automated sequencer ABI3100 (Macrogen Inc., Korea) to perform fragment analysis, in conjunction with MEGABACE FRAGMENT PROFILER v.1.2 software (Amersham Biosciences) and GENEIOUS v.6.0 (Drummond et al., 2009). Two researchers analyzed fragments independently, and in case of differences in allele calling, a consensus was reached by repeating amplification.

For fecal and museum samples, we built a consensus genotype based on the results of independent PCRs, following Frantz et al. (2003): a heterozygous genotype was assigned when supported by at least two PCRs yielding the same result, whereas a homozygous genotype was assigned after obtaining the same result in three PCRs. We performed downstream analyses including only samples successfully genotyped for at least seven out of 11 markers.

### 2.2 Distribution of genetic variability

We used ARLEQUIN v.3.5.1.2 (Excoffier et al., 2005) to calculate observed (*H*_o_) and expected heterozygosity (*H*_e_) for each marker. Using the same program, we checked for deviations from Hardy–Weinberg (H–W) equilibrium of expected frequencies (*α* = .01) and for signs of linkage disequilibrium (Bonferroni adjusted *α* = .0002). In case of linkage between markers, we eliminated the least informative one based on its theoretical probability of identity (*P*_ID_; Paetkau et al., 1995), calculated using GIMLET v.1.3.3 (Valière, 2002).

We assessed the overall pattern of genetic diversity through the genetic Landscape Shape Interpolation Analysis implemented by ALLELES IN SPACE v.1.0 (Miller, 2005). For this analysis, the software creates a connectivity network among individuals, calculates pairwise genetic distance between them, assigns this value to the geographical midpoint between individuals, and interpolates the value of genetic distance to the empty cells. We carried out this analysis using both connectivity networks created by ALLELES IN SPACE (pairwise of all observations and Delaunay) and calculating both raw genetic distance and its derived residual distance (which corrects for the effect of geographic distance). Using the same program, we ran a Mantel test between geographic and genetic distance with 10,000 permutations.

We conducted a spatial principal component analysis (sPCA) using the package adegenet in R v.4.0.3 (Jombart, 2008, 2015; R Core Team, 2020) with a Delaunay triangulation as the connectivity network. In this spatially explicit multivariate analysis, eigenvalues of principal components (PCs) are composite, measuring both variance and spatial autocorrelation. Negative eigenvalues represent local structures, while positive eigenvalues represent global structures (positive autocorrelation, i.e., isolation by distance [IBD] pattern or genetic clusters). This analysis does not assume H–W equilibrium or linkage equilibrium between loci, therefore complementing the broadly used Bayesian clustering methods. To decide which PCs to interpret, we analyzed the barplot of eigenvalues and the screeplot (i.e., decomposition of eigenvalues into their variance and spatial autocorrelation components) in conjunction with a global and local Monte‐Carlo test (with 9999 permutations) as done by Jombart et al. (2008). We repeated the analysis using a distance‐based connectivity network, where the maximum distance between points was long enough for all samples to have at least one neighbor, and short enough to minimize the number of connections over water.

### 2.3 Bayesian clustering analyses

Temporal and spatial bias in sampling schemes can have strong effects on the inference of population structure (Schwartz & McKelvey, 2009). Our samples spanned over a long period of time, a pattern commonly found in studies of carnivores where some samples are opportunistically obtained over multiple generations whereas others come from intense research efforts representing a few generations (Schwartz & McKelvey, 2009). To assess the possibility of temporal bias creating an artifact in our clustering results, we conducted Bayesian clustering methods using both the total number of samples (i.e., historical and contemporary samples) and contemporary samples only, comparing the results. For both datasets, we ran STRUCTURE v.2.3.4 (Pritchard et al., 2000) from *K* (number of clusters) = 1–10, 10 runs per *K*, and a burn‐in period of 500,000 steps followed by 1 million Markov chain Monte Carlo (MCMC) repetitions. We used an admixture model and correlated allele frequencies, with all remaining parameters on default settings. We summarized results using STRUCTURE HARVESTER v.0.6.94 (Earl & VonHoldt, 2012), and assessed convergence of the different runs for the same *K* using CLUMPAK (Kopelman et al., 2015). CLUMPAK also averaged the estimated cluster membership from the multiple runs. We selected the grouping scenario that best fitted our data by analyzing the mean log probability and Evanno's Δ*K* (Evanno et al., 2005).

IBD can create spurious genetic clustering of individuals into discrete units (Blair et al., 2012; Schwartz & McKelvey, 2009; Serre & Pääbo, 2004). Therefore, we repeated the analysis in STRUCTURE eliminating ≈25% of the samples representing the far north and far south locations, and compared these results with the ones previously obtained when using samples from the entire study area.

We used the spatial Bayesian clustering approach implemented by TESS v.2.3.1 (Chen et al., 2007), which incorporates geographic coordinates of samples as priors. Once again, we performed this analysis with all samples and contemporary samples only. In order to decide which admixture model to use (i.e., CAR described by Chen et al., 2007 or BYM proposed by Durand et al., 2009), we ran both models for the two data sets, setting *K* from 2 to 20, 10 runs per *K*, a burn‐in of 4000 sweeps, and 24,000 total number of sweeps, leaving the other settings on default values. Having decided on the admixture model, we performed the definitive runs for both data sets, from *K* = 2 to 10, 10 runs per *K*, a burn‐in of 51,000 sweeps and 300,000 as the total number of sweeps, leaving the other settings on default values. We averaged the estimated cluster membership from the multiple TESS runs using CLUMPAK.

### 2.4 Deviation from isolation by distance

Because Geoffroy's cat possesses a large distribution in relation to the species' dispersal capacity, the possibility of a global IBD pattern is likely. However, local deviations from this global pattern can occur when populations show differences in their dispersal due, for example, to differences in landscape resistance to animal movements (Kimmig et al., 2020). To assess this feature, we employed the Estimated Effective Migration Surfaces (EEMS) method (Petkova et al., 2016), which makes use of individual‐based migration rates to visualize areas with higher or lower migration with respect to the overall rate. This graphical method uses georeferenced samples to identify areas of abrupt genetic discontinuities in data sets with IBD patterns. We used the EEMS script for microsatellite analysis (runems_sats, available from https://github.com/dipetkov/eems) to create EEMS surfaces in R v.4.0.3. We performed the analyses using both the total number of samples and contemporary samples only. In addition, we ran the EEMS after removing samples collected at the far north and far south locations. To run this analysis, the study area was first divided into a grid where each vertex represented a deme, and individuals were assigned to the deme closest to their sampling location. We ran the model using different values for the number of demes present (see below for details). Then we computed a matrix of effective migration rates based on the stepping‐stone model. For the analysis with all samples and with only contemporary samples, we produced the final EEMS surfaces by averaging runs with 200, 400, and 600 demes, whereas for the trimmed dataset without extreme locations, we averaged runs with 50, 100, 200, 300, and 400 demes. Each run consisted of 1 million burn‐in steps followed by 20 million MCMC iterations sampled every 10,000 steps. As recommended by Petkova et al. (2016), the variance of the proposed distribution was adjusted for both migration and diversity parameters to ensure that all parameters were accepted between 20% and 30% of the time. We plotted the averaged EEMS and used the rEEMSplots package (Petkova et al., 2016) in R v.4.0.3 to check for MCMC convergence.

### 2.5 Cluster‐specific analyses

Using ARLEQUIN v.3.5.1.2, we calculated Ho and He in each inferred cluster and the genetic distance between clusters by computing the pairwise fixation index *F*_ST_. We estimated allelic richness in each cluster using the rarefaction method implemented in HP‐RARE v.1.1 (Kalinowski, 2005). We ran a Mantel test for each cluster using ALLELES IN SPACE with 10,000 permutations to explore the possibility of IBD pattern inside each cluster. The measure of genetic distance calculated by this software is the same one used by Nei et al. (1983), but adapted to pairs of individuals instead of pairs of populations (see Miller, 2005 for details). Given that this adapted measure does not make use of the population allele frequencies, we were concerned that our Mantel test could fail to detect subtle IBD patterns in each cluster. Therefore, we also performed a Mantel test using pairwise relatedness (QuellerGT estimator; Queller & Goodnight, 1989) as a measure of genetic similarity between pairs of individuals. We calculated pairwise relatedness with COANCESTRY v.1.0.1.9 (Wang, 2011), and performed a Mantel test between this estimator and geographic distance, using the R package ecodist (Goslee & Urban, 2007) with 10,000 permutations.

### 2.6 Niche differentiation

To evaluate if genetic structure is associated with different ecological niches, we built a genetic ecological niche model (gENM) for each cluster detected with Bayesian clustering methods (Ikeda et al., 2017; Milanesi et al., 2018). We downloaded all 19 standard climatic variables for current conditions from WorldClim v.2 (http://www.worldclim.org; 10 arcmin resolution; Fick & Hijmans, 2017), which represent the average based on records for the period 1970–2000 (see Appendix S1 for a description of each climatic variable).

First, we used the package Nichetoolbox (Osorio‐Olvera et al., 2020) in R v.4.0.3 to select the variables that summarize environmental information of each cluster (Pearson's correlation coefficient *r* < .85). Then we used WALLACE v.1.0.6.3 (Kass et al., 2018, 2023) in R to create ENMs. We performed a manual thinning of our presence data to reduce the effects of sampling bias by eliminating occurrence points if they were deemed to be too close to another point. We performed this subsampling of our occurrence data for each genetic cluster based on the resolution of climatic variables (10 arc min ≈ 340 km^2^ ≈ 18.5 km) as threshold, keeping only one point when two or more were separated by <18.5 km. We set the background extent to the entire Geoffroy's cat distribution with a 1‐degree buffer distance, 10,000 background points, and block (*k* = 4) spatial partitioning for model training and testing. We built models using the MaxEnt algorithm (Phillips et al., 2017), with feature classes L (linear), LQ (linear quadratic), H (Hinge), and LQH (linear quadratic hinge), with 0.5–5 regularization multipliers and the multiplier step value set to 0.5 (40 models). We chose the best model for each genetic cluster using a sequential criterion on cross‐validation results and the Akaike Information Criterion (AIC) score. Primarily, models were arranged by low omission rate (OR, 10 percentile training presence threshold). Because several models presented the same value, we chose the one with the lowest AIC score.

We evaluated possible climatic niche differentiation between genetic clusters using ENMTools package (Warren et al., 2021) in R. First, we measured the predicted climatic niche overlap between genetic clusters by calculating Schoener's *D* (Schoener, 1968) and Warren's *I* statistic (Warren et al., 2008). These measures range from 0 (i.e., niche models have no overlap) to 1 (i.e., niche models are identical). Based on Rödder and Engler (2011), we considered the following ranges to interpret intermediate results: 0–0.20 = no or very limited overlap; 0.21–0.40 = low overlap; 0.41–0.60 = moderate overlap; 0.61–0.80 = high overlap; and 0.81–1 = very high overlap. Also using ENMTools, we performed an identity test (Warren et al., 2008) to evaluate whether genetic clusters had significantly different niches. This test uses the *D* and *I* statistics calculated with empirical data, and compares them to an expected null distribution of values. The null distribution is built by: (1) randomizing the identity (i.e., the genetic cluster) attributed to each occurrence point while keeping the sample size of each genetic cluster consistent with empirical data, (2) using those points to create a niche model for each genetic cluster, (3) calculating *D* and *I* values of niche overlap, and (4) repeating the process n times to create a null distribution for *D* and *I*. We used the default settings (99 replicates) to create the null distribution, and compared it to the empirical *D* and *I* values. If empirical values fall inside the 95% confidence interval of the null distribution, the null hypothesis that niche models are identical should not be rejected.

The identity test does not take into account the environmental conditions that are available to each of the groups being compared, only real occurrence points. Groups inhabiting partially or fully allopatric regions could have distinct climatic niches due to differences in habitat availability, and not due to differences in habitat selection patterns or ecological preferences. Therefore, we conducted a Symmetrical Background test also available in ENMTools package, which evaluates if two groups are more similar/different than expected given the environmental conditions available to them (i.e., their environmental background). The test takes empirical measures of niche overlap (*D* and *I*) between two groups, and compares them to a null distribution of values representing overlap between niches calculated using random points across the range of each group. We used the default settings (99 replicates) to create a null distribution for *D* and for *I*, and compared them to the empirical *D* and *I* values. If empirical values fall inside the 95% confidence limits of the null distribution, the null hypothesis of background similarity should not be rejected. Rejection of the null hypothesis indicates that the niche models of two groups are more similar/different than expected by chance, given the existing differences between environments in their respective distribution ranges. Therefore, an observed niche differentiation between genetic clusters can be considered to be a product of differences in habitat preference and/or suitability between groups, and not an artifact due to environmental differences between habitats available to the groups (Warren et al., 2008).

Finally, we assessed if the maintenance of a niche boundary between clusters could be explained by the existence of a sharp environmental gradient (Glor & Warren, 2011). We ran a linear and a blob range‐breaking tests (LRB and BRB, respectively) using ENMTools to determine whether the climates of the geographic areas occupied were significantly different from each other (Glor & Warren, 2011), due to a steep environmental gradient. In LRB, the package creates a randomly drawn line (i.e., linear boundary) in the geographic range shared by both groups, splitting occurrence points into two artificial groups with sample sizes equal to the empirical data. Then, the package creates an ecological niche model for each newly generated group and measures overlap between them. This procedure is repeated n times to create a null distribution of overlap statistics between groups separated by a random linear barrier. If the empirical niche overlap statistics between groups fall outside the 95% confidence interval of the null distribution, it means the real barrier between groups creates a separation into environmental regions that are more different than expected by chance. The BRB test is similar to LRB, but instead of randomly drawing linear barriers to create artificial groups, it selects one occurrence point, assigns it to a group, and assigns all neighboring points to the same group until sample size is equal to empirical data. The rest of the test proceeds as in LRB: creation of a niche model for each artificial group, calculation of niche overlap statistics, and repetition to create a null distribution. For each test, we compared both empirical measures of niche overlap calculated previously (Schoener's *D* and Warren's *I*) against null distributions for each measure built from 100 randomized replicates.

## 3 RESULTS

### 3.1 Distribution of genetic variability

We successfully genotyped 135 samples (95.1% of the total samples extracted), representing 125 contemporary samples and 10 historical samples. Seven out of 11 markers showed deviations from H–W equilibrium proportions, in all cases due to heterozygote deficiency (Table S1). Markers FCA441 and F124 showed evidence of linkage, but the former had higher *P*_ID_, and therefore we discarded it from the rest of the analyses (*P*_ID_ for FCA441 = 1.605e‐01, *P*_ID_ for F126 = 4.178e‐02).

The pattern of genetic differences obtained with the Landscape Shape Interpolation analysis was the same using either raw or residual genetic distances, and there were only slight differences between the results from Delaunay and pairwise networks (Figure S1 shows results using raw genetic differences). In both cases, there was a north–south decrease in genetic differences between pairs of individuals. Mantel test showed a significant correlation between geographic and genetic distance (*p*‐value < .0001).

**FIGURE 2:**
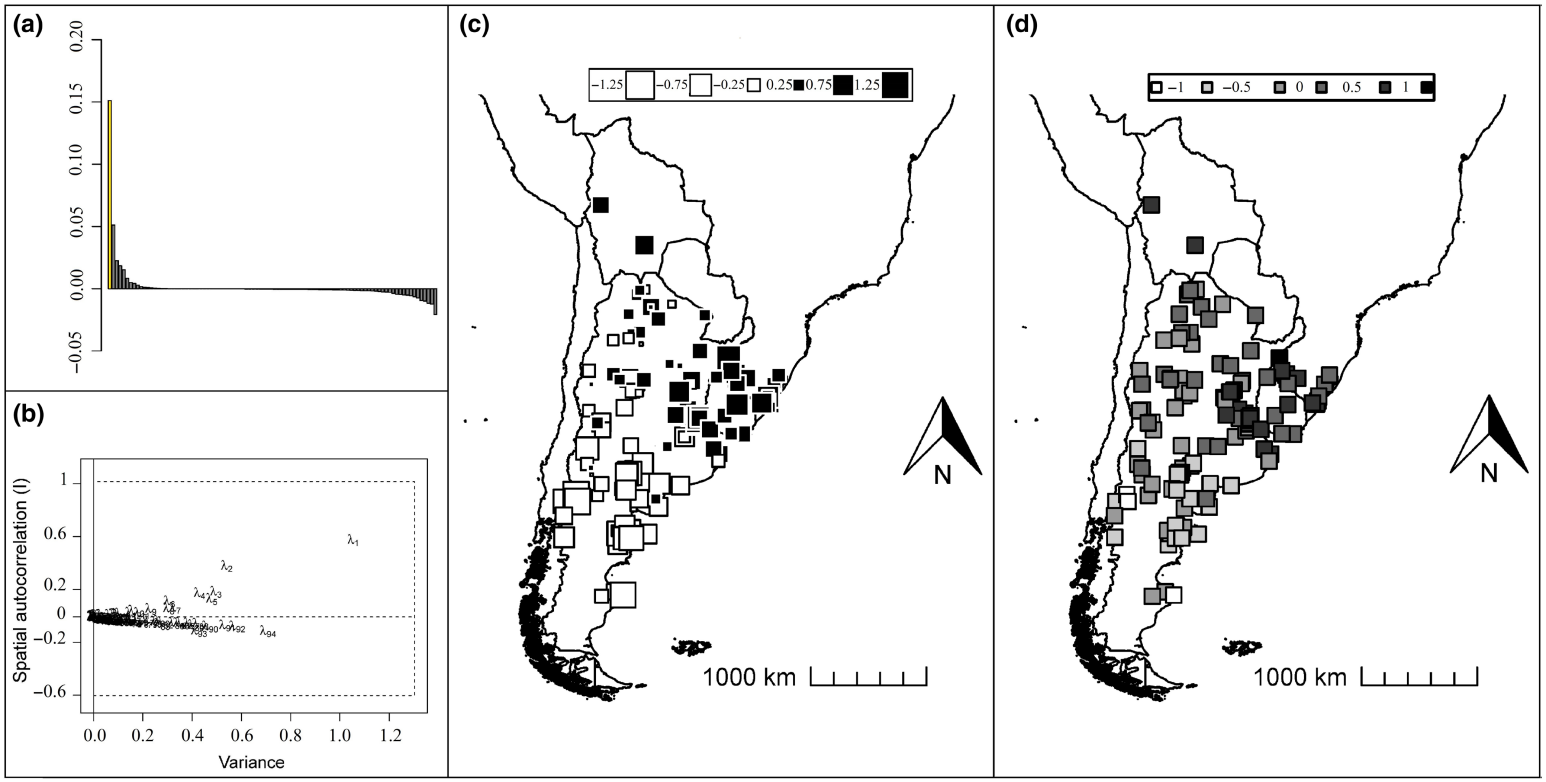
Results of the spatial principal component analysis obtained for samples of Geoffroy's cat (*Leopardus geoffroyi*), using a distance‐based connectivity network: (a) barplot of eigenvalues (in yellow: Principal component that we analyzed); (b) screeplot of the eigenvalues' decomposition; (c) values of the samples along the first principal component, plotted showing their geographical position, with larger white squares representing lower assigned values and larger black squares represent higher assigned values; (d) values of the samples along the first principal component, plotted showing their geographical position, with values represented in grayscale.

Monte‐Carlo tests with sPCA results revealed presence of global structure (*p*‐value = .0001) but no local structure (*p*‐value = .998) for both connectivity networks. The eigenvalues barplot and screeplot suggested that only the first principal component should be interpreted for both connectivity networks (Figure 2a,b show the results obtained with our distance‐based connectivity network). As shown in Figure 2c, the values of the samples along this first axis presented a clear pattern dividing the samples into north‐northeast and south‐southwest, roughly along the mid‐section of Argentina, and there was no need to clarify the pattern using the lag vectors. The values of the samples along the first principal component are also presented in grayscale (Figure 2d).

### 3.2 Bayesian clustering analyses

**FIGURE 3:**
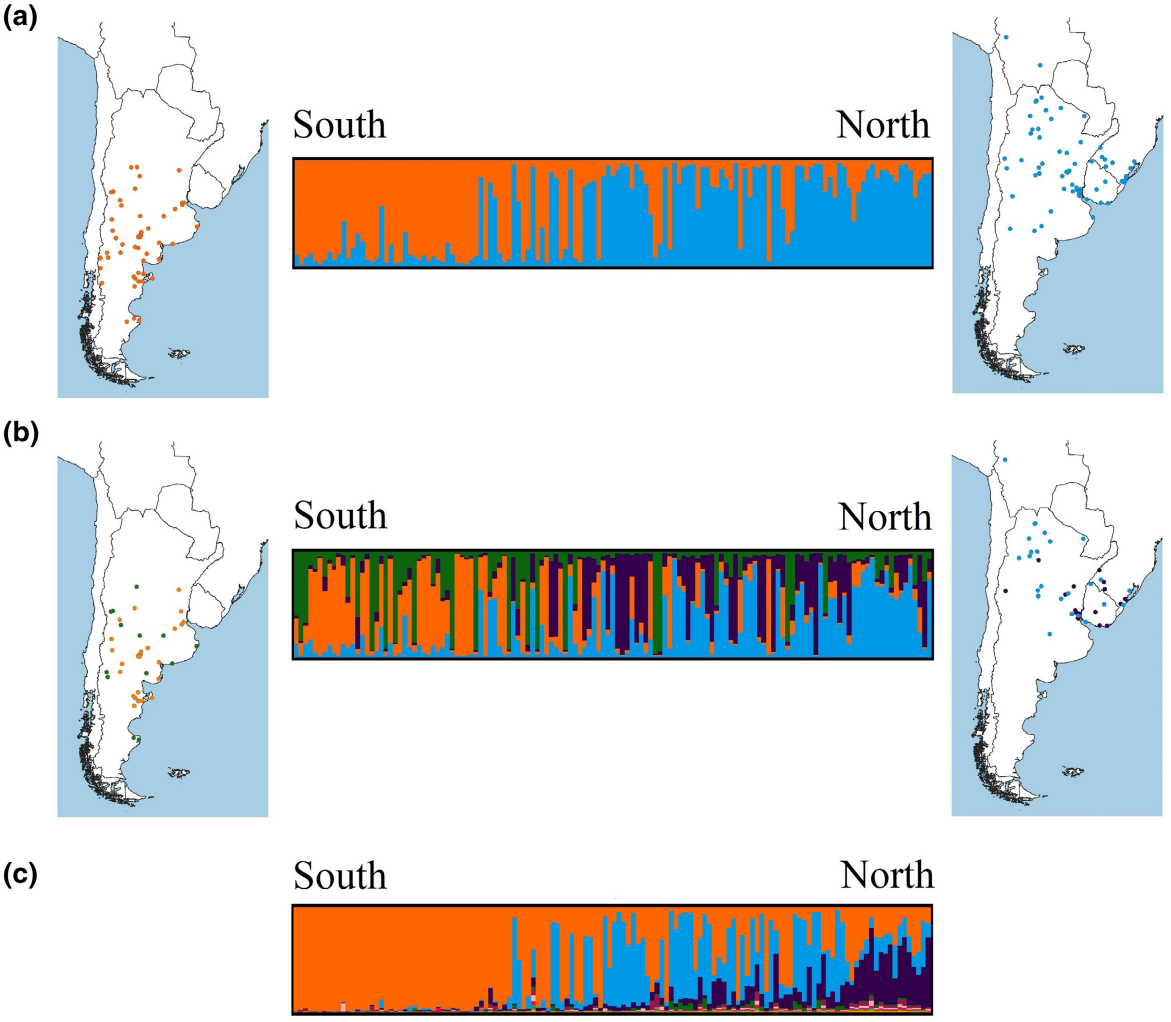
Results of the Bayesian clustering programs representing membership of Geoffroy's cats (*Leopardus geoffroyi*) to the different genetic clusters inferred. (a) Average STRUCTURE barplot obtained for *K* = 2 using all samples in our dataset (arranged in a south–north fashion), and their representation on a map (only samples with a membership value >0.7). (b) Average STRUCTURE barplot obtained for *K* = 4 using all samples in our dataset (arranged in a south–north fashion), and their representation on a map (only samples with a membership value >0.7). (c) Average TESS barplot obtained for *K*_max_ = 9 using all samples in our dataset (arranged in a south–north fashion). All maps were created using QGIS v.3.8.1‐Zanzibar (QGIS Development Team, 2018).

The results obtained using the whole dataset (historical and contemporary samples) were practically identical to the results obtained using contemporary samples alone, both in STRUCTURE and in TESS (Figure S2). Therefore, we decided to continue the analyses using the complete data set. STRUCTURE mean log probability suggested grouping the samples into four clusters, while according to Evanno's Δ*K* there could be 2–4 clusters (Figure S2). Individual memberships for *K* = 2 showed a clear relationship with geographic position (Figure 3a, minimum *Q* to assign membership = 0.7): one cluster in the north‐northeast section of the study area (*n* = 68; hereafter “CN”) and another in the south‐southwest (*n* = 57; hereafter “CS”), a pattern highly consistent with that obtained in the sPCA. The results for *K* = 4 represented a subdivision of each of these two clusters; however, this genetic structure did not encompass a geographical pattern (Figure 3b), as this solution would require sustained gene flow between distant areas (from the coast to the Andes) while avoiding gene flow with neighboring individuals.

**FIGURE 4:**
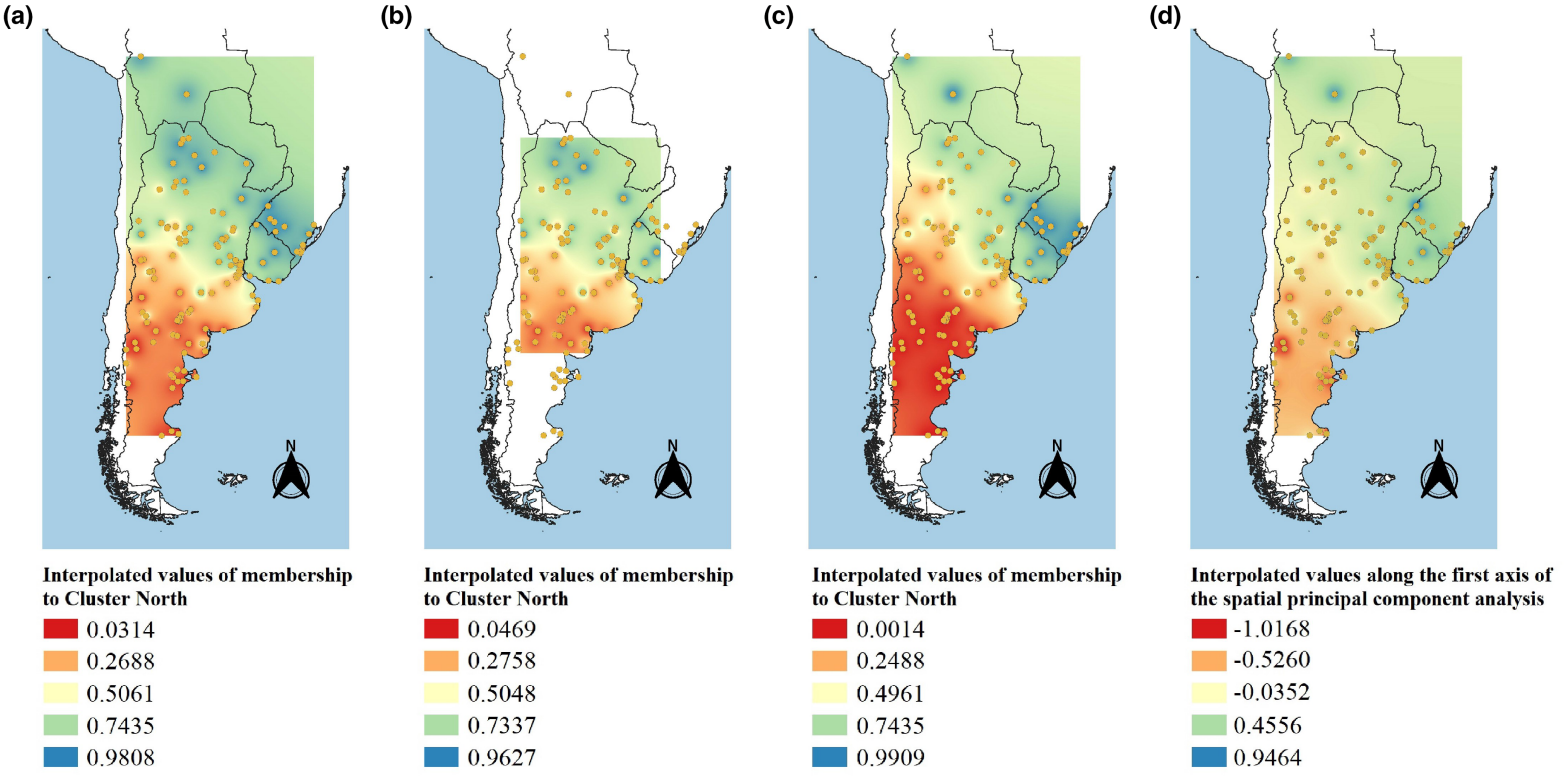
Interpolation of membership values of Geoffroy's cat (*Leopardus geoffroyi*) samples to inferred clusters (a) STRUCTURE (*K* = 2), all samples. (b) STRUCTURE (*K* = 2), without samples from extreme geographical positions. (c) TESS (*K* = 2), all samples. (d) Spatial principal component analysis, all samples. Color references for figures (a–c) blue represents high membership values to the northern cluster; red represents low membership value to the northern cluster (i.e., high membership values to the southern cluster). Color reference for figure (d) blue represents higher values along the first principal component; red represents lower values along the first principal component. All maps were created using QGIS v. 3.8.1‐Zanzibar (QGIS Development Team, 2018).

The lowest Deviance Information Criterion for TESS results was obtained at *K*_max_ = 9. However, the averaged membership under this model only showed two of these clusters being represented by our samples (Figure 3c; minimum *Q* to assign membership = 0.7). The average barplot for *K* = 2 was characterized by a membership distribution that closely resembled the clusters found with STRUCTURE and the spatial pattern obtained through sPCA, with one cluster in the north‐northeast and another in the south‐southwest. Figure 4 shows the results of the Bayesian analyses and sPCA as interpolations, to allow for easy comparison between different methods. As shown in the maps, *K* = 2 produced almost identical results for both Bayesian clustering methods; therefore, we decided to focus on explaining genetic structure at the *K* = 2 level, instead of *K* = 4, which was only supported by STRUCTURE. This decision is also in alignment with STRUCTURE software manual, which recommends interpreting the smallest *K* in cases where similar values of Pr(*X*|*K*) are obtained for multiple *K*s (Pritchard et al., 2010).

The same grouping pattern was inferred when running STRUCTURE after removing samples with more extreme geographical positions, supporting the idea that inferred genetic structure is not an artifact due to the presence of IBD (Figure 4a,b).

### 3.3 Deviation from isolation by distance

**FIGURE 5:**
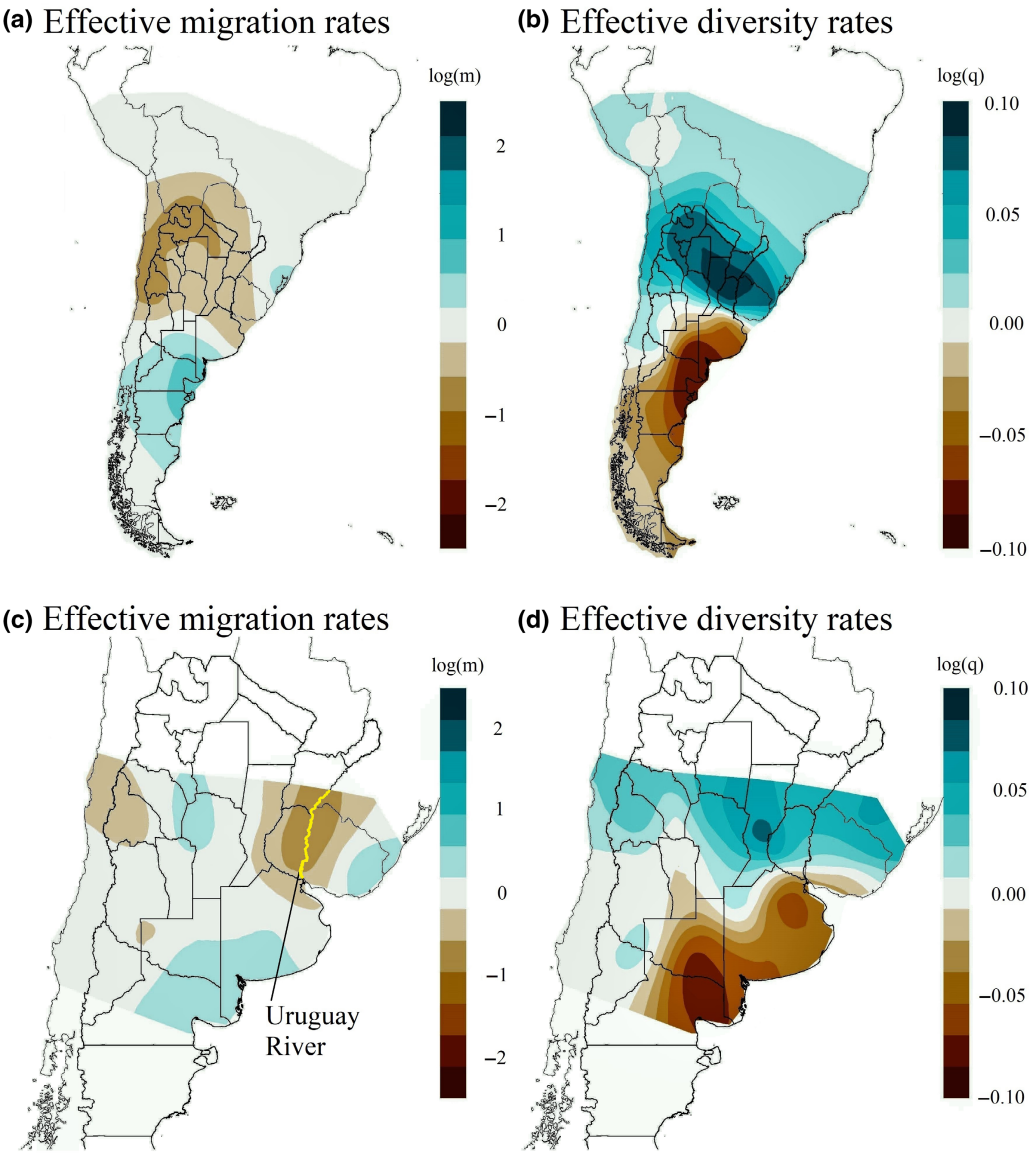
Individual‐based estimated effective migration surfaces analysis of effective migration rates (*m*) and diversity rates (*q*) for Geoffroy's cats. Panels (a, b) represent the whole dataset, while panels (c, d) represent trimmed dataset after removing samples from extreme north and extreme south locations. Panels (a, c) show the interpolated surface of the posterior mean m (on a log_10_ scale) representing deviations from continuous gene flow. Darker ochre areas have lower than expected effective gene flow, while darker blue areas have higher than expected gene flow. Panels (b, d) show the interpolated surface of the posterior mean *q* (on a log_10_ scale) representing effective diversity across the study area. Darker ochre areas have lower than average diversity, while darker blue areas have higher than average diversity. Uruguay River is shown in yellow, panel (c).

The individual‐based effective migration surfaces produced for each dataset using EEMS are shown in Figure 5. As in the case of Bayesian clustering, the results were practically identical using either the complete data set (historical and contemporary samples) or contemporary samples alone, and thus we only present the results based on the complete dataset. We obtained a similar pattern to the Landscape Shape Interpolation analysis, with lower rates of migration and higher rates of diversity in the northern area of Geoffroy's cat distribution. On the contrary, the southern area displays higher rates of migration and lower diversity rates. The pattern also resembles the two clusters found with STRUCTURE and TESS (Figure 4a–c).

The trimmed dataset showed a more local migration pattern, with low migration rates in the northeast region comprising southwestern Brazil, western Uruguay, and a portion of eastern Argentina. Two additional small areas with limited migration were observed toward the west (Figure 5c). We found no deviation of IBD in the center of the species distribution, but because sampling was low in this area, some local patterns could be missing. On the other hand, the genetic diversity pattern of the trimmed dataset was similar to the one obtained for the complete dataset.

### 3.4 Cluster‐specific analyses

The *F*_ST_ between clusters was low to moderate (0.052) but significant (*p*‐value < .00001). The CS showed lower rarefied allelic richness than CN for all loci except for F42 (Table 1). Locus FCA424 only showed two alleles in CN, while appearing monomorphic in CS (Table 1). Mantel test between geographic and genetic distances as calculated in Alleles in Space was significant for CN (*r* = .124; *p*‐value = .001) but not for CS (*r* = .081; *p*‐value = .083). On the other hand, the Mantel test performed using relatedness as a measure of genetic similarity was significant for both clusters, albeit more pronounced in CN (*r* = −.142; *p*‐value < .001) than in CS (*r* = −.101; *p* = .009).

**TABLE 1:** Locus‐specific assessments in each cluster inferred for Geoffroy's cat: Observed heterozygosity (*H*_o_), expected heterozygosity (*H*_e_) under H–W equilibrium, statistical significance of the difference between *H*_o_ and *H*_e_ (*p*‐value, statistically significant results in bold), and rarefied allelic richness (AR_rar_).

Locus	CN				CS			
	*H*_o_	*H*_e_	*p*‐Value	AR_rar_	*H*_o_	*H*_e_	*p*‐Value	AR_rar_
FCA742	0.765	0.866	**.009**	12.131	0.649	0.748	.052	5.863
FCA391	0.647	0.726	.257	7.857	0.667	0.684	.175	4.000
F53	0.618	0.859	**<.001**	13.765	0.561	0.830	**<.001**	8.754
FCA723	0.600	0.951	**<.001**	22.595	0.640	0.922	**<.001**	17.000
F146	0.824	0.713	.289	7.661	0.368	0.491	**<.001**	4.863
F42	0.836	0.886	.579	10.731	0.807	0.869	.146	11.872
FCA424	0.176	0.231	.079	2.000	Monomorphic			1.000
FCA453	0.652	0.751	.110	5.942	0.786	0.731	.357	5.882
F124	0.677	0.835	**<.001**	9.717	0.596	0.717	**<.001**	5.876
F98	0.333	0.389	.074	3.000	0.140	0.192	.098	2.877
*Mean*	*0.612*	*0.720*		*9.540*	*0.579*	*0.687*		*6.800*

*Note*: Locus FCA441 was eliminated from the analysis because it showed signs of linkage with locus F124 and had higher probability of identity. *Italic*: Mean values.

Abbreviations: CN, Cluster North; CS, Cluster South.

### 3.5 Niche differentiation

**FIGURE 6:**
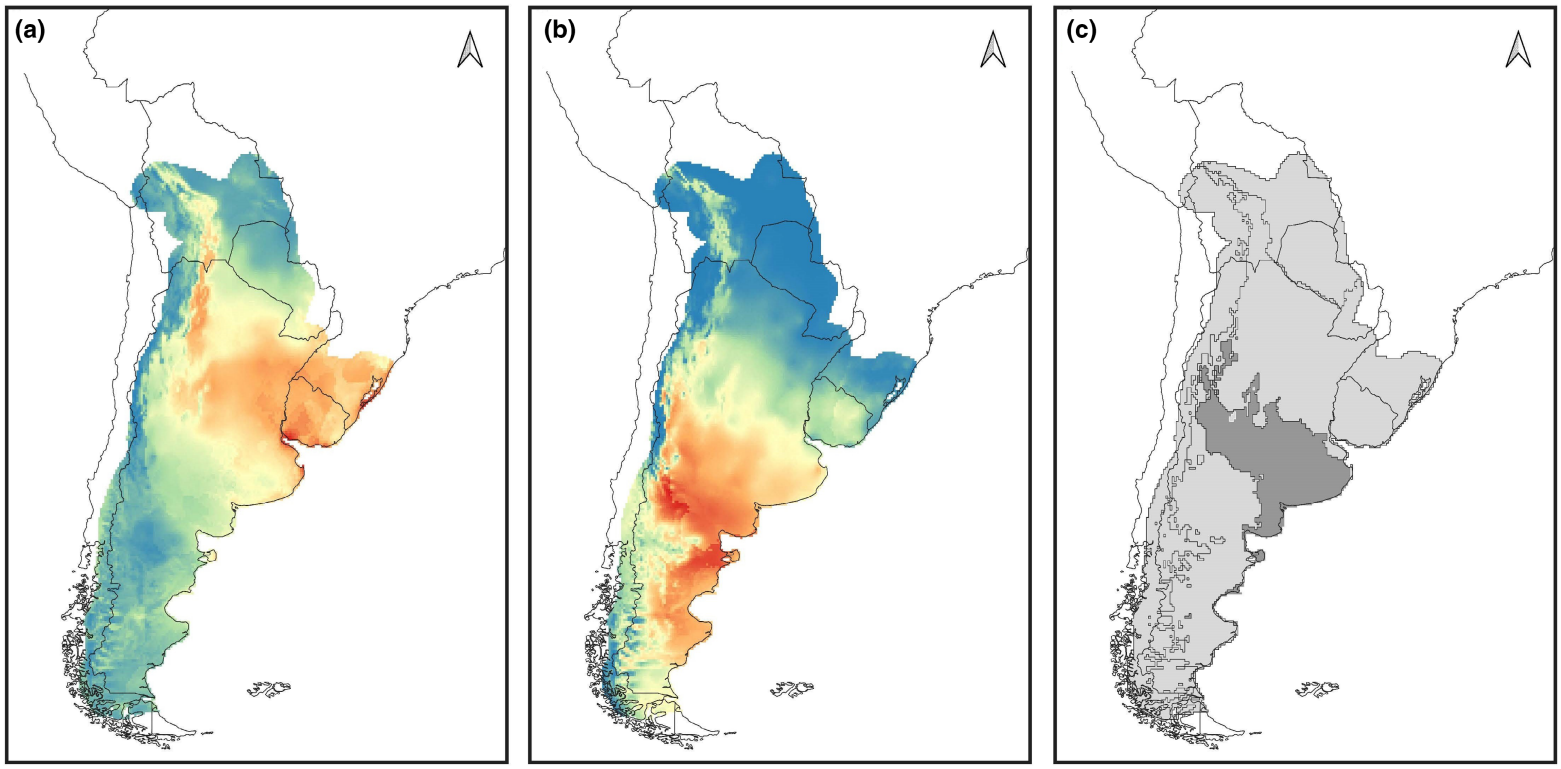
MaxEnt results of ecological niche models (run using Wallace 1.0.6.3) showing habitat suitability of genetic clusters inferred for Geoffroy's cat (*Leopardus geoffroyi*): (a) Cluster North (CN) and (b) Cluster South (CS). Blue areas indicate lower predicted suitability, while red areas indicate higher predicted suitability. (c) Map displaying the overlapping of high suitability for CN and for CS (i.e., area where predicted suitability values were above 50% for both genetic clusters), represented in darker gray.

For each cluster, 10 bioclimatic variables were regarded as having low pairwise correlation (Pearson's correlation coefficient *r* < .85). We used these 10 variables to build the gENMs for each cluster (Table S2), six of which were shared by both clusters (Bio01, Bio02, Bio03, Bio09, Bio12, and Bio14). The best supported model for each cluster according to the OR and AIC is shown in Figure 6. For CN, highest suitability areas for Geoffroy's cat were located in the northeastern part of the species range including central and eastern Argentina, Uruguay, and southern Brazil (Figure 6a). Highest suitability areas for CS, on the other hand, were distributed over central and southeastern Argentina (Figure 6b). The geographic overlap of suitability between clusters, defined as the geographic area where both clusters had a predicted suitability >50% (not to be confused with niche overlap), occurred in an extensive zone of central Argentina (Figure 6c). The area under the curve (AUC) values indicated good to high model performances (AUC_CS_ = 0.82; AUC_CN_ = 0.71).

Out of the 10 bioclimatic variables used to build the gENMs, seven were retained in the best supported model of each cluster, with three variables shared between clusters (Table 2, Figures S3 and S4). For CN, increases in the mean temperature of the wettest quarter (Bio08) correlated with an increase in suitability up to a point in which further increases in Bio08 did not increase suitability. Isothermality (Bio03) and precipitation of the wettest month (Bio13) showed moderate changes in suitability at lower values of the variables, after which suitability remained relatively high (>0.65). Medium values of temperature seasonality (Bio04) predicted maximum suitability, which dropped sharply with further increases or decreases of this variable (Figure S3).

**TABLE 2:** Bioclimatic variables contributing to the most accurate niche model for each cluster inferred for Geoffroy's cat (*Leopardus geoffroyi*) in this work (see Figures S3 and S4).

CN	CS	Gómez Fernández et al. (2020)^a^	Cuyckens et al. (2016)^b^
**Bio01 = Annual Mean Temperature** (↑↓)	**Bio01 = Annual Mean Temperature** (↑)	Bio04 = Temperature Seasonality	Bio04 = Temperature Seasonality (↑)
**Bio02 = Mean Diurnal Range** (↓₋)	**Bio02 = Mean Diurnal Range** (↑)	Bio06 = Min Temperature of Coldest Month	Bio06 = Min Temperature of Coldest Month (↑↓)
Bio03 = Isothermality (↓₋)	Bio06 = Min Temperature of Coldest Month (↑↓)	Bio13 = Precipitation of Wettest Month	Bio12 = Annual Precipitation (↑⎺↓⎽)
Bio04 = Temperature Seasonality (↑↓)	Bio09 = Mean Temperature of Driest Quarter (↑)	Bio15 = Precipitation Seasonality	
Bio08 = Mean Temperature of Wettest Quarter (↑⎺)	**Bio14 = Precipitation of Driest Month** (↓)	Bio17 = Precipitation of Driest Quarter	
Bio13 = Precipitation of Wettest Month (↑⎺)	Bio15 = Precipitation Seasonality (↓)		
**Bio14 = Precipitation of Driest Month** (↑⎺)	Bio18 = Precipitation of Warmest Quarter (↓)		

*Note*: We also present bioclimatic variables contributing to the niche model found for the entire species distribution in previous studies, which treated the species as genetically uniform and having a single environmental niche. In bold, variables shared between genetic clusters. The symbols in parentheses represent a simplified description of the relationship between the values of a variable and the predicted suitability for the species: ↑ = increasing values of the variable result in increasing suitability; ↓ = increasing values of the variable result in decreasing suitability; ↑↓ = increasing values of the variable result in increasing suitability up to a point where further increases in the variable result in decreased suitability; ↑⎺ = increasing values of the variable result in increasing suitability up to a point where suitability remains stable; ↓⎽ = increasing values of the variable result in decreasing suitability up to a point where suitability remains stable.

Abbreviations: CN, Cluster North; CS, Cluster South.

^a^ Gómez Fernández et al. (2020) used 72 samples and 266 presence records, representing the same area covered in our study, from southern Patagonia to Bolivia and southern Brazil.

^b^ Cuyckens et al. (2016) used 1502 presence records covering an area similar to the one in our study, with records spanning from southern Patagonia to Bolivia and southern Brazil, but unlike our study, the authors included records from Paraguay.

For CS, predicted climatic suitability decreased as precipitation seasonality (Bio15) and precipitation of the warmest quarter (Bio18) increased, but it showed a constant positive relationship as mean temperature of the driest quarter (Bio09) increased. Minimum temperature of the coldest month (Bio06) predicted maximum suitability near 0°C, dropping sharply with further increases or decreases of this climatic variable (Figure S3).

The three environmental variables shared between CN and CS displayed different responses for each cluster (Figure S4). For CN, annual mean temperature (Bio01) showed a positive relationship with suitability at lower temperatures, reaching a maximum at 17°C–20°C then rapidly decreasing after this point (Figure S4, top). For CS, this variable showed a constant positive relationship with suitability (i.e., increasing suitability with higher temperatures), reaching its highest point at near 26°C. The response curve for Mean Diurnal Range (Bio02) showed for CN the highest suitability at lowest values of this variable, then declining toward middle ranges and remaining constant after approx. 10.5°C. For CS, in turn, suitability slightly increased as the mean diurnal range increased (Figure S4, middle). Finally, precipitation of the driest month (Bio14) predicted for CN an abrupt gain in suitability of approx. 24% at low precipitation values (5–10 mm), remaining constant at the highest suitability values (approx. 0.78) after that point. For CS, suitability decreased as precipitation of the driest month increased (Figure S4, bottom).

Based on both measures of niche overlap, there was moderate to high niche overlap between CN and CS (*D* = 0.471, *I* = 0.739) based on Rödder and Engler (2011) range values. However, the identity tests showed these niches were significantly different (*p*‐value = .01, Figure S5a), with both empirical *D* and *I* statistics showing that niches overlap less than expected based on the null distribution, and the null hypothesis of identical niche was rejected. In the same way, symmetrical background tests demonstrated that niches are less similar than expected by chance between clusters considering their environmental availability (*p*‐value = .001; Figure S5b). Supporting these results, both the linear and blob range‐break tests indicated that environmental divergence of the geographic areas occupied were significantly different from each other (LRB *p*‐value = .001, BRB *p*‐value = .03; Figure S5c,d), suggesting the influence of a steep environmental gradient separating genetic clusters, creating a division where environments at each side of the barrier are more different than expected by chance.

## 4 DISCUSSION

We found two Geoffroy's cat distinct genetic clusters throughout the species range, with one cluster occupying the north‐northeast part of the species distribution and the other in the south‐southwest. These clusters occupy different environmental niches, and this difference is not explained by habitat availability in the area occupied by each cluster. This indicates that individuals belonging to each cluster have affinity for different climate conditions (or the resources that these conditions make possible), supporting the idea that ENMs might generate less realistic results when they assume that species are genetically uniform and have a single environmental niche (Gotelli & Stanton‐Geddes, 2015).

As is the case in studies inferring climatic niches (for examples studying neotropical felids specifically, see Arias‐Alzate et al., 2017; Charre‐Medellín et al., 2023; Cuyckens et al., 2016; Espinosa et al., 2018; Gómez Fernández et al., 2020; Sartor et al., 2021), the climatic variables contributing significantly to the models could be affecting the species' distribution directly, indirectly, or through a combination of both. For example, the climatic variables found to affect habitat suitability for Geoffroy's cat could be doing so by affecting vegetation community and influencing distribution of cats' prey species, which in turn would influence cats' distribution. Models including species interactions (e.g., parasites, predators, prey, and competitors) are extremely complex, and it was not our goal to determine these interactions. Regardless of whether climatic variables are influencing distribution patterns of cats directly (e.g., through seasonal floods) or indirectly (e.g., affecting vegetation or prey availability), we can conclude which variables affect Geoffroy's cat distribution, and we can estimate what conditions determine habitat suitability for each genetic cluster inferred.

All clustering analyses performed recovered the genetic partition into two clusters, geographically consistent with sPCA results, but the occurrence of such partition was unexpected given the ecological plasticity of this felid and its ability to disperse long distances even through human‐dominated landscapes (Pereira & Novaro, 2014; Sartor et al., 2022). STRUCTURE suggested the possibility of subdivision of each of the main clusters into two, but the existence of such pattern would require areas separated by long distances to be somehow connected by enough gene flow to remain genetically similar as the same genetic unit. At the same time, individuals dispersing those long distances would have to avoid mating with nearby individuals from the other sub‐cluster. Therefore, we focused on the *K* = 2 solution, which was supported by STRUCTURE and our other analyses. We found a significant global pattern of IBD, as well as within each cluster, suggesting that a portion of the observed population differentiation may be explained by the dispersal capabilities of Geoffroy's cat. The EEMS uncovered a pattern of lower dispersal rates in the northern cluster and higher rates to the south; therefore, other processes (e.g., landscape resistance, isolation by barriers), which can lead to similar results in Mantel test (Cushman & Landguth, 2010; Ruiz‐Gonzalez et al., 2015), could be generating the differentiation between groups. The structure we found could be a combination of several processes, and therefore exploring alternative causal factors would be the next step.

Bou et al. (2021) examined Geoffroy's cat population structure and gene flow in the Uruguayan Savanna ecoregion encompassing Uruguay, southeastern Brazil, and a portion of eastern Argentina, and found two distinct genetic clusters with an *F*_ST_ value between clusters (0.042) similar to the estimated in our study (0.052). These authors suggested that genetic differences may reflect landscape heterogeneities and the role of the Uruguay River (see Figure 5c) as a substantial natural barrier for the species movement, a pattern also observed by Sartor et al. (2022). In our study, although no obvious physical barrier to gene flow occurs, low migration rates were found for the same area, as shown in Figure 5c. In addition, the existence of a steep environmental gradient affecting gene flow is supported by the linear and blob range‐break tests. This steep difference could be ascribed to the contact zone between clusters being located in an environmental gradient known as the Arid Diagonal, which in turn leads to the area subjected to the highest level of anthropogenic land modification in South America (Kennedy et al., 2019, 2020; Figure 1b), the Argentine Pampas ecoregion. The anthropogenic disturbance in this ecoregion began at the end of the 19th century through farming intervention, but a new phase characterized by the intensification of agricultural practices, initiated in the late 1980s, lead to a massive transformation of natural habitats (Aizen et al., 2009; Baldi & Paruelo, 2008; Paruelo et al., 2006). Accompanying this agricultural intensification process, a highly connected road network was developed, including several of the most important main roads of Argentina. A genetics‐based optimized landscape resistance model for Geoffroy's cat developed by Sartor et al. (2022) showed that agricultural areas do not impose high resistance to movement for Geoffroy's cat per se, but the almost complete removal of the natural vegetation due to agricultural intensification strongly increases resistance to movement for this species. In addition, main roads were also found to impose substantial resistance for Geoffroy's cat movements. Accordingly, Sartor et al. (2022) found some of the highest landscape resistance values for the current Geoffroy's cat genetic connectivity in the Pampas ecoregion, the contact area between the two genetic clusters inferred in our study. Thus, recent human‐induced habitat modifications (i.e., occurred during the last century) may have driven current population genetic differentiation in this widely distributed species. The observed pattern, unraveled through analysis of microsatellite markers, differs from the genetic structure found through the analysis of mitochondrial DNA (Gómez Fernández et al., 2020), reflecting that historical biogeographical processes and recent human‐induced effects have contributed differently to shape the extant population genetic structure of Geoffroy's cat.

Geographical variation in population genetic diversity is also influenced by both historical and contemporary changes to population size and gene flow (Vucetich & Waite, 2003), and thus the phylogeographical study of Gómez Fernández et al. (2020) likely provides a valuable framework to evaluate historical influences on the contemporary genetic diversity of Geoffroy's cat. The origin of this small felid is located in Central Argentina, from where the lineage diversification spread toward the north, east, and west reaching Paraguay, Bolivia, Uruguay, Brazil, and the Andes. Finally, the last lineage diversification arrived ca. 20 kya to southern Argentina and Chile, during the Last Glacial Maximum, and resulted in the presence of Geoffroy's cat in the far south of Patagonia (Gómez Fernández et al., 2020). In our study, the highest genetic diversity (i.e., allelic richness) was found in the northern cluster, which encloses the geographical origin of the species and the more diverse clade (Gómez Fernández et al., 2020). The southern cluster exhibited the lowest genetic diversity although it showed the highest mobility. An explanation for this genetic impoverishment toward the south could be the occurrence of sequential founder events during the range expansion after glaciations. When Patagonia became suitable habitat for the species, the expansion occurred through a north–south colonization route, and the allelic richness would have been reduced as population grew from a relatively small number of founders (Haanes et al., 2010; Hampe & Petit, 2005). In a review of the genetic variation across species' geographical ranges based on nuclear molecular markers, Eckert et al. (2008) showed that central populations usually sustain higher genetic diversity than peripheral populations, and our findings agree with this globally observed pattern.

On the other hand, contemporary factors may also have contributed to the impoverished genetic diversity estimated toward the south of the species distribution. By 1980, Geoffroy's cat became the most traded felid in the world (MacMahan, 1986), and a large proportion of pelts destined to the fur trade originated in southern Argentina (J. A. Pereira, personal communication). Although the legal trade dropped after 1986, the massive removal of individuals may have contributed to decimating the remaining populations, with consequent effects on allelic diversity. A decrease in allelic richness and heterozygosity has been observed in other carnivore species subjected to intense persecution (e.g., *Canis lupus*; Flagstad et al., 2003). Although our study included both historical and contemporary samples, the small number of historical samples prevented us from directly testing the possible effect of intense hunting on the genetic diversity in the southern genetic cluster. Since poaching and predation by domestic dogs are still the main mortality causes affecting this felid in southern Argentina (e.g., Pereira et al., 2010), human‐related mortality could be currently contributing to maintain the low genetic diversity observed in the southern part of the species range.

Besides the observed north–south gradient in allelic richness, the genetic diversity estimated in our work (*H*_e_: 0.689) was slightly lower than the estimated by other authors for the whole Geoffroy's cat distribution (*H*_e_ = 0.748 in Johnson et al., 1999; *H*_e_ = 0.732 in Sartor et al., 2021) and even for particular areas within the species distribution (*H*_e_ = 0.740 in Tirelli et al., 2018; *H*_e_ = 0.714 in Trigo et al., 2008; *H*_e_ = 0.770 in Bou et al., 2021). These differences could be attributed to disparities in sampling sizes among studies or to the geographical distribution of samples, since the studies used for comparison were mostly (i.e., >80%) based on samples originated at the northern half of the Geoffroy's cat range. Thus, the lower heterozygosity obtained in our study likely reflects the inclusion of a large proportion of samples from the southern edge of the species range, where genetic diversity is impoverished.

We found moderate to high ecological niche overlap between the two clusters. However, results from the Niche identity and Symmetrical Background tests were consistent with climate niche divergence, indicating that each genetic cluster occurred in different climate conditions. Because species distributions are influenced by both climate and genetics, incorporating genetic structure into the species–climate relationship will lead to more accurate ENMs, especially for species with broad and environmentally heterogeneous distributions such as Geoffroy's cat (Ikeda et al., 2017). Therefore, when we compared our results with niche models that consider the entire distribution (Cuyckens et al., 2016; Gómez Fernández et al., 2020), we observed differences in the areas of highest suitability and the bioclimatic features that influence the presence of Geoffroy's cat (Table 2). The main difference is that each genetic cluster shows areas of low suitability where higher values were previously predicted when assessing suitability for the species as a single unit (i.e., global models). For example, the highest suitability in global models was in the center of the distribution, but CS values were low in this area. In addition, the bioclimatic variables that make up the global models were distributed among the genetic ENMs obtained for each cluster. However, none of these variables was shared between the two clusters (Table 2).

The bioclimatic variables that summarized the environmental information for the CN were mostly related with variation in temperature, whereas both temperature and precipitation showed comparable influences for the CS. In addition, the different responses to the three bioclimatic predictors shared between clusters could be showing the plasticity of the species that allows them to adapt to different environmental conditions.

For CS, the predicted suitability decreased as Precipitation of the warmest quarter and Precipitation of the driest month increased, suggesting a species adaptation to the arid and semiarid conditions present on the distribution range of this cluster. Furthermore, the observed response curves of CS for variables shared between clusters were in some cases inverse to the responses observed for CN (Table 2). These differences, coupled with lower genetic variability in CS and the restrictions to gene flow evidenced by the genetic structure, could mean that the individuals of the southern cluster are under different pressures, maybe produced by the particular dry habitat present at the southern part of South America. Because there is no straightforward connection between neutral and adaptive genetic differentiation (Reed & Frankham, 2003), it is likely that the differences between the ecological niches of genetic clusters may be due to the plasticity of the species (Guidobono et al., 2016; Pereira & Aprile, 2012) combined with the high hunting pressure experienced by the species. Further studies analyzing potential links between adaptive genetic markers within each cluster and environmental features could help to fully unravel the difference found.

Our study indicates that further conservation measures to protect Geoffroy's cat should take into account the genetic structure we inferred. The steeper response curves observed during the niche analysis for CS suggest that this genetic cluster is more vulnerable to the effects of the current context of climate change. A more abundant precipitation in summer and a thermal warming trend have been observed or projected for Patagonia and the Monte region (Labraga & Villalba, 2009; Solomon et al., 2007), the two most represented ecoregions across the CS distribution range. Thus, adaptation measures (i.e., the identification of practical strategies to reduce anticipated effects of climate change) would be required to conserve populations within CS (e.g., Pereira & Novaro, 2014). Additional measures focused on conserving both genetic clusters should be helpful to maintain the evolutionary potential of the species as a whole in the face of the ongoing global change.

## AUTHOR CONTRIBUTIONS

**Alberto F. Fameli:** Data curation (equal); formal analysis (equal); investigation (equal); methodology (equal); visualization (equal); writing – original draft (lead); writing – review and editing (equal). **Javier A. Pereira:** Conceptualization (equal); data curation (equal); funding acquisition (lead); investigation (equal); project administration (equal); resources (lead); supervision (equal); writing – review and editing (equal). **Julio Rojo Gómez:** Conceptualization (equal); data curation (equal); investigation (equal); methodology (supporting); writing – review and editing (equal). **María Jimena Gómez Fernández:** Conceptualization (equal); data curation (equal); formal analysis (equal); investigation (equal); methodology (equal); project administration (supporting); supervision (lead); visualization (equal); writing – review and editing (equal).

## FUNDING INFORMATION

Funding was provided by FONCyT (PICT 2016‐4087), CONICET (PIP 114‐201101‐00050), Amersfoort Zoo, Feline Conservation Federation, and Parc des Félins. Capture and sampling permits by Disposición DNCAP N°12/07 (APN), Disposición 26/08 (APN), Disposición 149/14 (Dirección de Flora y Fauna, Ministerio de Asuntos Agrarios, Provincia de Buenos Aires).

## CONFLICT OF INTEREST STATEMENT

The authors declare no conflicts of interest.

## Supporting information


Appendix S1.

## Data Availability

Data is archived in Dryad in “Private for peer review” status under doi: 10.5061/dryad.9s4mw6mpc, and can be accessed using the following link: https://datadryad.org/stash/share/u31_5Qq4YRFDUPQAAMhPj6DkuB2dKxCV2A4ALDiahuY.

## References

[ece370223-bib-0001] Aizen, M. A. , Garibaldi, L. A. , & Dondo, M. (2009). Expansión de la soja y diversidad de la agricultura Argentina. Ecología Austral, 19(1), 45–54.

[ece370223-bib-0002] Arias‐Alzate, A. , González‐Maya, J. F. , Arroyo‐Cabrales, J. , & Martínez‐Meyer, E. (2017). Wild felid range shift due to climatic constraints in the Americas: A bottleneck explanation for extinct felids? Journal of Mammalian Evolution, 24, 427–438.

[ece370223-bib-0003] Baldi, G. , & Paruelo, J. M. (2008). Land‐use and land cover dynamics in South American temperate grasslands. Ecology and Society, 13(2), [online] URL: http://www.ecologyandsociety.org/vol13/iss2/art6/

[ece370223-bib-0004] Blair, C. , Weigel, D. E. , Balazik, M. , Keeley, A. T. , Walker, F. M. , Landguth, E. , Cushman, S. , Murphy, M. , Waits, L. , & Balkenhol, N. (2012). A simulation‐based evaluation of methods for inferring linear barriers to gene flow. Molecular Ecology Resources, 12(5), 822–883. 22551194 10.1111/j.1755-0998.2012.03151.x

[ece370223-bib-0005] Bou, N. , Soutullo, Á. , Hernández, D. , Mannise, N. , González, S. , Bartesaghi, L. , Pereira, J. , Merino, M. , Espinosa, C. , Trigo, T. C. , & Cosse, M. (2021). Population structure and gene flow of Geoffroy's cat (*Leopardus geoffroyi*) in the Uruguayan Savanna ecoregion. Journal of Mammalogy, 102(3), 879–890.

[ece370223-bib-0006] Boutin‐Ganache, I. , Raposo, M. , Raymond, M. , & Deschepper, C. F. (2001). M13‐tailed primers improve the readability and usability of microsatellite analyses performed with two different allele‐sizing methods. BioTechniques, 31(1), 25–28. 11464515

[ece370223-bib-0007] Broad, S. (1987). The harvest of and trade in Latin American spotted cats (Felidae) and otters (Lutrinae). Wildlife Trade Monitoring Unit.

[ece370223-bib-0008] Canepuccia, A. D. , Farias, A. A. , Escalante, A. H. , Iribarne, O. , Novaro, A. , & Isacch, J. P. (2008). Differential responses of marsh predators to rainfall‐induced habitat loss and subsequent variations in prey availability. Canadian Journal of Zoology, 86(5), 407–418.

[ece370223-bib-0009] Castillo, D. , Luengos Vidal, E. , Lucherini, M. , & Casanave, E. B. (2008). First report on the Geoffroy's cat in a highly modified rural area of the Argentine pampas. Cat News, 49, 27–28.

[ece370223-bib-0010] Charre‐Medellín, J. F. , Ferrer‐Ferrando, D. , Monterrubio‐Rico, T. C. , Fernández‐López, J. , & Acevedo, P. (2023). Using species distribution modeling to generate relative abundance information in socio‐politically unstable territories: Conservation of Felidae in the central‐western region of Mexico. Ecology and Evolution, 13(9), e10534. 37727774 10.1002/ece3.10534 PMC10505758

[ece370223-bib-0011] Chen, C. , Durand, E. , Forbes, F. , & François, O. (2007). Bayesian clustering algorithms ascertaining spatial population structure: A new computer program and a comparison study. Molecular Ecology Notes, 7(5), 747–756.

[ece370223-bib-0012] Coates, D. J. , Byrne, M. , & Moritz, C. (2018). Genetic diversity and conservation units: Dealing with the species‐population continuum in the age of genomics. Frontiers in Ecology and Evolution, 6, 165.

[ece370223-bib-0013] Crandall, K. A. , Bininda‐Emonds, O. R. , Mace, G. M. , & Wayne, R. K. (2000). Considering evolutionary processes in conservation biology. Trends in Ecology & Evolution, 15(7), 290–295. 10856956 10.1016/s0169-5347(00)01876-0

[ece370223-bib-0014] Cushman, S. A. , & Landguth, E. L. (2010). Spurious correlations and inference in landscape genetics. Molecular Ecology, 19(17), 3592–3602. 20618896 10.1111/j.1365-294X.2010.04656.x

[ece370223-bib-0015] Cuyckens, G. A. E. , Pereira, J. A. , Trigo, T. C. , Da Silva, M. , Gonçalves, L. , Huaranca, J. C. , Bou Pérez, N. , Cartes, J. L. , & Eizirik, E. (2016). Refined assessment of the geographic distribution of Geoffroy's cat (*Leopardus geoffroyi*)(Mammalia: Felidae) in the Neotropics. Journal of Zoology, 298(4), 285–292.

[ece370223-bib-0016] Drummond, A. J. , Ashton, B. , Buxton, S. , Cheung, M. , Cooper, A. , Duran, C. , Field, M. , Heled, J. , Kearse, M. , Markowitz, S. , Moir, R. , Stones‐Havas, S. , Sturrock, S. , Thierer, T. , & Wilson, A. (2009). Geneious Pro v47 .

[ece370223-bib-0017] Durand, E. , Jay, F. , Gaggiotti, O. E. , & François, O. (2009). Spatial inference of admixture proportions and secondary contact zones. Molecular Biology and Evolution, 26(9), 1963–1973. 19461114 10.1093/molbev/msp106

[ece370223-bib-0018] Earl, D. A. , & VonHoldt, B. M. (2012). STRUCTURE HARVESTER: A website and program for visualizing STRUCTURE output and implementing the Evanno method. Conservation Genetics Resources, 4, 359–361.

[ece370223-bib-0019] Eckert, C. G. , Samis, K. E. , & Lougheed, S. C. (2008). Genetic variation across species' geographical ranges: The central‐marginal hypothesis and beyond. Molecular Ecology, 17(5), 1170–1188. 18302683 10.1111/j.1365-294X.2007.03659.x

[ece370223-bib-0020] Espinosa, C. C. , Trigo, T. C. , Tirelli, F. P. , da Silva, L. G. , Eizirik, E. , Queirolo, D. , Mazim, F. D. , Peters, F. B. , Favarini, M. O. , & de Freitas, T. R. (2018). Geographic distribution modeling of the margay (*Leopardus wiedii*) and jaguarundi (*Puma yagouaroundi*): A comparative assessment. Journal of Mammalogy, 99(1), 252–262.

[ece370223-bib-0021] Evanno, G. , Regnaut, S. , & Goudet, J. (2005). Detecting the number of clusters of individuals using the software STRUCTURE: A simulation study. Molecular Ecology, 14(8), 2611–2620. 15969739 10.1111/j.1365-294X.2005.02553.x

[ece370223-bib-0022] Excoffier, L. , Laval, G. , & Schneider, S. (2005). Arlequin (version 3.0): An integrated software package for population genetics data analysis. Evolutionary Bioinformatics Online, 1, 47–50. PMC2658868 19325852

[ece370223-bib-0023] Fick, S. E. , & Hijmans, R. J. (2017). WorldClim 2: New 1‐km spatial resolution climate surfaces for global land areas. International Journal of Climatology, 37(12), 4302–4315.

[ece370223-bib-0024] Flagstad, Ø. , Walker, C. W. , Vilà, C. , Sundqvist, A. K. , Fernholm, B. , Hufthammer, A. K. , Wiig, Ø. , Koyola, I. , & Ellegren, H. (2003). Two centuries of the Scandinavian wolf population: Patterns of genetic variability and migration during an era of dramatic decline. Molecular Ecology, 12(4), 869–880. 12753208 10.1046/j.1365-294x.2003.01784.x

[ece370223-bib-0025] Frantz, A. C. , Pope, L. C. , Carpenter, P. J. , Roper, T. J. , Wilson, G. J. , Delahay, R. J. , & Burke, T. (2003). Reliable microsatellite genotyping of the Eurasian badger (*Meles meles*) using faecal DNA. Molecular Ecology, 12(6), 1649–1661. 12755892 10.1046/j.1365-294x.2003.01848.x

[ece370223-bib-0026] Glor, R. E. , & Warren, D. (2011). Testing ecological explanations for biogeographic boundaries. Evolution, 65(3), 673–683. 21054358 10.1111/j.1558-5646.2010.01177.x

[ece370223-bib-0027] Gómez Fernández, M. J. , Fameli, A. , Rojo Gómez, J. , Pereira, J. A. , & Mirol, P. (2020). Phylogeographical spatial diffusion analysis reveals the journey of Geoffroy's cat through the quaternary glaciations of South America. Biological Journal of the Linnean Society, 129(3), 603–617.

[ece370223-bib-0028] Goslee, S. C. , & Urban, D. L. (2007). The ecodist package for dissimilarity‐based analysis of ecological data. Journal of Statistical Software, 22, 1–19.

[ece370223-bib-0029] Gotelli, N. J. , & Stanton‐Geddes, J. (2015). Climate change, genetic markers and species distribution modelling. Journal of Biogeography, 42(9), 1577–1585.

[ece370223-bib-0030] Guidobono, J. S. , Muñoz, J. , Muschetto, E. , Teta, P. , & Busch, M. (2016). Food habits of Geoffroy's cat (*Leopardus geoffroyi*) in agroecosystem habitats of Buenos Aires, Argentina. Ecología Austral, 26(1), 40–50.

[ece370223-bib-0031] Guisan, A. , & Thuiller, W. (2005). Predicting species distribution: Offering more than simple habitat models. Ecology Letters, 8(9), 993–1009. 34517687 10.1111/j.1461-0248.2005.00792.x

[ece370223-bib-0032] Haanes, H. , Røed, K. H. , Flagstad, Ø. , & Rosef, O. (2010). Genetic structure in an expanding cervid population after population reduction. Conservation Genetics, 11, 11–20.

[ece370223-bib-0033] Hampe, A. , & Petit, R. J. (2005). Conserving biodiversity under climate change: The rear edge matters. Ecology Letters, 8(5), 461–467. 21352449 10.1111/j.1461-0248.2005.00739.x

[ece370223-bib-0034] Ikeda, D. H. , Max, T. L. , Allan, G. J. , Lau, M. K. , Shuster, S. M. , & Whitham, T. G. (2017). Genetically informed ecological niche models improve climate change predictions. Global Change Biology, 23, 164–176. 27543682 10.1111/gcb.13470

[ece370223-bib-0035] Johnson, W. E. , & Franklin, W. L. (1991). Feeding and spatial ecology of *Felis geoffroyi* in southern Patagonia. Journal of Mammalogy, 72(4), 815–820.

[ece370223-bib-0036] Johnson, W. E. , Slattery, J. P. , Eizirik, E. , Kim, J. H. , Raymond, M. M. , Bonacic, C. , Cambre, R. , Crawshaw, P. , Nunes, A. , Seuánez, H. N. , Moreira, M. A. , Seymour, K. L. , Simon, F. , Swanson, W. , & O'Brien, S. J. (1999). Disparate phylogeographic patterns of molecular genetic variation in four closely related south American small cat species. Molecular Ecology, 8, S79–S94. 10703553 10.1046/j.1365-294x.1999.00796.x

[ece370223-bib-0037] Jombart, T. (2008). Adegenet: A R package for the multivariate analysis of genetic markers. Bioinformatics, 24(11), 1403–1405. 18397895 10.1093/bioinformatics/btn129

[ece370223-bib-0038] Jombart, T. (2015). A tutorial for the spatial analysis of principal components (sPCA) using adegenet 2.1.0. MRC Centre for Outbreak Analysis and Modelling, Imperial College London.

[ece370223-bib-0039] Jombart, T. , Devillard, S. , Dufour, A. B. , & Pontier, D. (2008). Revealing cryptic spatial patterns in genetic variability by a new multivariate method. Heredity, 101(1), 92–103. 18446182 10.1038/hdy.2008.34

[ece370223-bib-0040] Kalinowski, S. T. (2005). Hp‐rare 1.0: A computer program for performing rarefaction on measures of allelic richness. Molecular Ecology Notes, 5(1), 187–189.

[ece370223-bib-0041] Kass, J. M. , Pinilla‐Buitrago, G. E. , Paz, A. , Johnson, B. A. , Grisales‐Betancur, V. , Meenan, S. I. , Attali, D. , Broennimann, O. , Galante, P. J. , Maitner, B. S. , Owens, H. L. , Varela, S. , Aiello‐Lammens, M. E. , Merow, C. , Blair, M. E. , & Anderson, R. P. (2023). Wallace 2: A shiny app for modeling species niches and distributions redesigned to facilitate expansion via module contributions. Ecography, 2023(3), e06547.

[ece370223-bib-0042] Kass, J. M. , Vilela, B. , Aiello‐Lammens, M. E. , Muscarella, R. , Merow, C. , & Anderson, R. P. (2018). Wallace: A flexible platform for reproducible modeling of species niches and distributions built for community expansion. Methods in Ecology and Evolution, 9(4), 1151–1156.

[ece370223-bib-0043] Kennedy, C. M. , Oakleaf, J. R. , Theobald, D. M. , Baruch‐Mordo, S. , & Kiesecker, J. (2019). Managing the middle: A shift in conservation priorities based on the global human modification gradient. Global Change Biology, 25(3), 811–826. 30629311 10.1111/gcb.14549

[ece370223-bib-0044] Kennedy, C. M. , Oakleaf, J. R. , Theobald, D. M. , Baruch‐Mordo, S. , & Kiesecker, J. (2020). Global human modification of terrestrial systems. NASA Socioeconomic Data and Applications Center (SEDAC). 10.7927/edbc-3z60

[ece370223-bib-0045] Kimmig, S. E. , Beninde, J. , Brandt, M. , Schleimer, A. , Kramer‐Schadt, S. , Hofer, H. , Börner, K. , Schulze, C. , Wittstatt, U. , Heddergott, M. , Halczok, T. , Staubach, C. , & Frantz, A. C. (2020). Beyond the landscape: Resistance modelling infers physical and behavioural gene flow barriers to a mobile carnivore across a metropolitan area. Molecular Ecology, 29(3), 466–484. 31880844 10.1111/mec.15345

[ece370223-bib-0046] Kopelman, N. M. , Mayzel, J. , Jakobsson, M. , Rosenberg, N. A. , & Mayrose, I. (2015). Clumpak: A program for identifying clustering modes and packaging population structure inferences across K. Molecular Ecology Resources, 15(5), 1179–1191. 25684545 10.1111/1755-0998.12387 PMC4534335

[ece370223-bib-0047] Labraga, J. C. , & Villalba, R. (2009). Climate in the Monte Desert: Past trends, present conditions, and future projections. Journal of Arid Environments, 73(2), 154–163.

[ece370223-bib-0048] MacMahan, L. R. (1986). The international cat trade. In S. D. Miller & D. D. Everett (Eds.), Cats of the world: Biology, conservation, and management (pp. 461–488). National Wildlife Federation.

[ece370223-bib-0049] Mares, M. A. , & Ojeda, R. A. (1984). Faunal commercialization and conservation in South America. Bioscience, 34(9), 580–584.

[ece370223-bib-0050] Menotti‐Raymond, M. , David, V. A. , Lyons, L. A. , Schäffer, A. A. , Tomlin, J. F. , Hutton, M. K. , & O'Brien, S. J. (1999). A genetic linkage map of microsatellites in the domestic cat (*Felis catus*). Genomics, 57(1), 9–23. 10191079 10.1006/geno.1999.5743

[ece370223-bib-0051] Menotti‐Raymond, M. A. , David, V. A. , Wachter, L. L. , Butler, J. M. , & O'Brien, S. J. (2005). An STR forensic typing system for genetic individualization of domestic cat (*Felis catus*) samples. Journal of Forensic Science, 50(5), 1061–1070. 16225210

[ece370223-bib-0052] Milanesi, P. , Caniglia, R. , Fabbri, E. , Puopolo, F. , Galaverni, M. , & Holderegger, R. (2018). Combining Bayesian genetic clustering and ecological niche modeling: Insights into wolf intraspecific genetic structure. Ecology and Evolution, 8(22), 11224–11234. 30519439 10.1002/ece3.4594 PMC6262746

[ece370223-bib-0053] Miller, M. P. (2005). Alleles in space (AIS): Computer software for the joint analysis of interindividual spatial and genetic information. Journal of Heredity, 96(6), 722–724. 16251514 10.1093/jhered/esi119

[ece370223-bib-0054] Moritz, C. (1994). Defining ‘evolutionarily significant units’ for conservation. Trends in Ecology & Evolution, 9(10), 373–375. 21236896 10.1016/0169-5347(94)90057-4

[ece370223-bib-0055] Nascimento, F. O. D. (2014). On the morphological variation and taxonomy of the Geoffroy's cat *Leopardus geoffroyi* (d'Orbigny & Gervais, 1844)(Carnivora, Felidae). Papéis Avulsos de Zoologia, 54, 129–160.

[ece370223-bib-0056] Nei, M. , Tajima, F. , & Tateno, Y. (1983). Accuracy of estimated phylogenetic trees from molecular data: II. Gene frequency data. Journal of Molecular Evolution, 19, 153–170. 6571220 10.1007/BF02300753

[ece370223-bib-0057] Nowell, K. , & Jackson, P. (1996). Wild cats: Status survey and conservation action plan. IUCN/SSC Cat Specialist Group. IUCN.

[ece370223-bib-0058] Osorio‐Olvera, L. , Lira‐Noriega, A. , Soberón, J. , Peterson, A. T. , Falconi, M. , Contreras‐Díaz, R. G. , Martínez‐Meyer, E. , Barve, V. , & Barve, N. (2020). Ntbox: An R package with graphical user interface for modelling and evaluating multidimensional ecological niches. Methods in Ecology and Evolution, 11(10), 1199–1206.

[ece370223-bib-0059] Paetkau, D. , Calvert, W. , Stirling, I. , & Strobeck, C. (1995). Microsatellite analysis of population structure in Canadian polar bears. Molecular Ecology, 4(3), 347–354. 7663752 10.1111/j.1365-294x.1995.tb00227.x

[ece370223-bib-0060] Paruelo, J. M. , Guerschman, J. P. , Pineiro, G. , Jobbagy, E. G. , Verón, S. R. , Baldi, G. , & Baeza, S. (2006). Cambios en el uso de la tierra en Argentina y Uruguay: Marcos conceptuales para su análisis. Agrociencia Uruguay, 10(2), 47–61.

[ece370223-bib-0061] Pereira, J. A. , & Aprile, G. (2012). Felinos de Sudamerica. Londaiz Laborde.

[ece370223-bib-0062] Pereira, J. A. , Fracassi, N. G. , Rago, V. , Ferreyra, H. , Marull, C. A. , McAloose, D. , & Uhart, M. M. (2010). Causes of mortality in a Geoffroy's cat population—A long‐term survey using diverse recording methods. European Journal of Wildlife Research, 56(6), 939–942.

[ece370223-bib-0063] Pereira, J. A. , Lucherini, M. , & Trigo, T. (2015). *Leopardus geoffroyi*. The IUCN Red List of Threatened Species 2015, e.T15310A50657011. https://www.iucnredlist.org/

[ece370223-bib-0064] Pereira, J. A. , & Novaro, A. J. (2014). Habitat‐specific demography and conservation of Geoffroy's cats in a human‐dominated landscape. Journal of Mammalogy, 95(5), 1025–1035.

[ece370223-bib-0065] Petkova, D. , Novembre, J. , & Stephens, M. (2016). Visualizing spatial population structure with estimated effective migration surfaces. Nature Genetics, 48(1), 94–100. 26642242 10.1038/ng.3464 PMC4696895

[ece370223-bib-0066] Phillips, S. J. , Anderson, R. P. , Dudík, M. , Schapire, R. E. , & Blair, M. E. (2017). Opening the black box: An open‐source release of Maxent. Ecography, 40(7), 887–893.

[ece370223-bib-0067] Pritchard, J. K. , Stephens, M. , & Donnelly, P. (2000). Inference of population structure using multilocus genotype data. Genetics, 155(2), 945–959. 10835412 10.1093/genetics/155.2.945 PMC1461096

[ece370223-bib-0068] Pritchard, J. K. , Wen, X. , & Falush, D. (2010). Documentation for structure software: Version 2.3 (pp. 1–37). University of Chicago.

[ece370223-bib-0069] QGIS Development Team . (2018). QGIS geographic information system . Open Source Geospatial Foundation Project. http://qgis.osgeo.org

[ece370223-bib-0070] Queller, D. C. , & Goodnight, K. F. (1989). Estimating relatedness using genetic markers. Evolution, 43(2), 258–275. 28568555 10.1111/j.1558-5646.1989.tb04226.x

[ece370223-bib-0071] R Core Team . (2020). R: A language and environment for statistical computing. R Foundation for Statistical Computing.

[ece370223-bib-0072] Reed, D. H. , & Frankham, R. (2003). Correlation between fitness and genetic diversity. Conservation Biology, 17(1), 230–237.

[ece370223-bib-0073] Rödder, D. , & Engler, J. O. (2011). Quantitative metrics of overlaps in Grinnellian niches: Advances and possible drawbacks. Global Ecology and Biogeography, 20(6), 915–927.

[ece370223-bib-0074] Ruiz‐Gonzalez, A. , Cushman, S. A. , Madeira, M. J. , Randi, E. , & Gómez‐Moliner, B. J. (2015). Isolation by distance, resistance and/or clusters? Lessons learned from a forest‐dwelling carnivore inhabiting a heterogeneous landscape. Molecular Ecology, 24(20), 5110–5129. 26394893 10.1111/mec.13392

[ece370223-bib-0075] Sartor, C. C. , Cushman, S. A. , Wan, H. Y. , Kretschmer, R. , Pereira, J. A. , Bou, N. , Cosse, M. , González, S. , Eizirik, E. , de Freitas, T. R. O. , & Trigo, T. C. (2021). The role of the environment in the spatial dynamics of an extensive hybrid zone between two Neotropical cats. Journal of Evolutionary Biology, 34(4), 614–627. 33484012 10.1111/jeb.13761

[ece370223-bib-0076] Sartor, C. C. , Wan, H. Y. , Pereira, J. A. , Eizirik, E. , Trigo, T. C. , de Freitas, T. R. O. , & Cushman, S. A. (2022). Landscape genetics outperforms habitat suitability in predicting landscape resistance for congeneric cat species. Journal of Biogeography, 49(12), 2206–2217.

[ece370223-bib-0077] Schoener, T. W. (1968). The Anolis lizards of Bimini: Resource partitioning in a complex fauna. Ecology, 49(4), 704–726.

[ece370223-bib-0078] Schwartz, M. K. , & McKelvey, K. S. (2009). Why sampling scheme matters: The effect of sampling scheme on landscape genetic results. Conservation Genetics, 10, 441–452.

[ece370223-bib-0079] Serre, D. , & Pääbo, S. (2004). Evidence for gradients of human genetic diversity within and among continents. Genome Research, 14(9), 1679–1685. 15342553 10.1101/gr.2529604 PMC515312

[ece370223-bib-0080] Solomon, S. , Qin, D. , Manning, M. , Marquis, M. , Averyt, K. , Tignor, M. M. B. , LeRoy Miller, H. L., Jr. , & Chen, Z. (2007). Climate change 2007: The physical science basis: Contribution of working group I to the fourth assessment report of the intergovernmental panel on climate change. Cambridge Univ. Press.

[ece370223-bib-0081] Tirelli, F. P. , Trigo, T. C. , Trinca, C. S. , Albano, A. P. N. , Mazim, F. D. , Queirolo, D. , Espinosa, C. C. , Soares, J. B. , Pereira, J. A. , Crawshaw, P. G. , Macdonald, D. W. , Lucherini, M. , & Eizirik, E. (2018). Spatial organization and social dynamics of Geoffroy's cat in the Brazilian pampas. Journal of Mammalogy, 99(4), 859–873.

[ece370223-bib-0082] Trigo, T. C. , Freitas, T. R. , Kunzler, G. , Cardoso, L. , Silva, J. C. , Johnson, W. E. , O'Brien, S. J. , Bonatto, S. L. , & Eizirik, E. (2008). Inter‐species hybridization among Neotropical cats of the genus *Leopardus*, and evidence for an introgressive hybrid zone between *L. geoffroyi* and *L. tigrinus* in southern Brazil. Molecular Ecology, 17(19), 4317–4333. 18785898 10.1111/j.1365-294X.2008.03919.x PMC6993176

[ece370223-bib-0083] Valière, N. (2002). GIMLET: A computer program for analysing genetic individual identification data. Molecular Ecology Notes, 2(3), 377–379.

[ece370223-bib-0084] Vucetich, J. A. , & Waite, T. A. (2003). Spatial patterns of demography and genetic processes across the species' range: Null hypotheses for landscape conservation genetics. Conservation Genetics, 4, 639–645.

[ece370223-bib-0085] Wang, J. (2011). COANCESTRY: A program for simulating, estimating and analysing relatedness and inbreeding coefficients. Molecular Ecology Resources, 11(1), 141–145. 21429111 10.1111/j.1755-0998.2010.02885.x

[ece370223-bib-0086] Warren, D. L. , Glor, R. E. , & Turelli, M. (2008). Environmental niche equivalency versus conservatism: Quantitative approaches to niche evolution. Evolution, 62(11), 2868–2883. 18752605 10.1111/j.1558-5646.2008.00482.x

[ece370223-bib-0087] Warren, D. L. , Matzke, N. J. , Cardillo, M. , Baumgartner, J. B. , Beaumont, L. J. , Turelli, M. , Glor, R. E. , Huron, N. A. , Simões, M. , Iglesias, T. L. , Piquet, J. C. , & Dinnage, R. (2021). ENMTools 1.0: An R package for comparative ecological biogeography. Ecography, 44(4), 504–511.

[ece370223-bib-0088] Ximénez, A. (1975). Felis geoffroyi. Mammalian Species, 54, 1–4.

