# Supplementary material for: Genetic structure and climate niche differentiation among populations of Leopardus geoffroyi
Source: Ecol Evol. 2024 Aug 30;14(9):e70223. doi: 10.1002/ece3.70223 (PMC11362614; doi:10.1002/ece3.70223)
Supplement: Supplementary file 1 — Appendix S1. [file ECE3-14-e70223-s001.docx]

Lab procedures

We used a panel of eleven microsatellite loci (F42, F53, F98, F124, F146, FCA391, FCA424, FCA441, FCA453, FCA723, FCA742) developed for domestic cats (Menotti-Raymond et al. 1999, 2005). PCR amplifications were performed individually using the M13-tailed primer method (Boutin-Ganache et al., 2001) to label amplicons with 5′-fluorescent tags (6-FAM, HEX or NED). For non-invasive samples, we followed the multiple tube approach according to Franz et al. (2003) to minimize genotyping errors. A consensus genotype was assigned to each sample after a minimum of two independent positive PCRs for heterozygotes, and three for homozygotes. We arranged PCR products in two multiplexes according to allele size and fluorescent dye. Each microsatellite amplification reaction included negative controls. Amplifications were carried out in a total volume of 12μl containing 1.8-2.2μl of DNA; 1X Taq buffer; (750mm Tris-HCl, 200mM (NH4)2SO4, 0.1% (v/v) Tween 20); 2.5-3.75mM of MgCl2; 0.08mM of each dNTP; 0.24 pmoles of M13-tailed forward primer; 4 pmoles of reverse primer, 6 pmoles of M13-fluorescent primer; 0.2mg/ml of BSA and 0.6U of Taq DNA polymerase (Fermentas). Thermocycling conditions were as follow: 94ºC for 5 min; 10 touchdown cycles of 94ºC for 40 s, 60-51ºC for 40 s and 72ºC for 60 s; 15 cycles of 94ºC for 40 s, (i) 53.4 ºC (markers F42, F124, F146, FCA391, FCA441, FCA723, FCA742), (ii) 52.5°C (markers F53, FCA453), (iii) 51.7°C (marker FCA424) or (iv) 50°C (marker F98) for 40 s, 72ºC for 60 s; 10 cycles of 94ºC for 40 s, 48ºC for 30 s, 72ºC for 40 s and a final extension at 72ºC for 5 min.

List of WorldClim variables

Bio01-Annual Mean Temperature

Bio02-Mean Diurnal Range: Mean of monthly (max temp - min temp)

Bio03-Isothermality (Bio02/Bio07) (×100)

Bio04-Temperature Seasonality (standard deviation ×100)

Bio05-Max Temperature of Warmest Month

Bio06-Min Temperature of Coldest Month

Bio07-Temperature Annual Range (Bio05-Bio06)

Bio08-Mean Temperature of Wettest Quarter

Bio09-Mean Temperature of Driest Quarter

Bio10-Mean Temperature of Warmest Quarter

Bio11-Mean Temperature of Coldest Quarter

Bio12-Annual Precipitation

Bio13-Precipitation of Wettest Month

Bio14-Precipitation of Driest Month

Bio15-Precipitation Seasonality (coefficient of variation)

Bio16-Precipitation of Wettest Quarter

Bio17-Precipitation of Driest Quarter

Bio18-Precipitation of Warmest Quarter

Bio19-Precipitation of Coldest Quarter.

Results

**Table S1** Locus-specific assessments of observed heterozygosity (Ho), expected heterozygosity (He) under H-W equilibrium, statistical significance of the difference between Ho and He (p-value, statistically significant results in bold, α=0.01), probability of identity (P_ID_).

^a^ We removed locus FCA441 from the analysis because it showed signs of linkage with locus F124.

| Locus | Ho | He | p-value | P_ID_ |
| --- | --- | --- | --- | --- |
| FCA742 | 0.726 | 0.827 | **0.000** | 5.41E-02 |
| FCA391 | 0.652 | 0.700 | 0.070 | 1.43E-01 |
| F53 | 0.600 | 0.857 | **0.000** | 3.83E-02 |
| FCA723 | 0.608 | 0.943 | **0.000** | 7.00E-03 |
| F146 | 0.612 | 0.671 | **0.001** | 1.62E-01 |
| F42 | 0.813 | 0.889 | 0.179 | 2.39E-02 |
| FCA424 | 0.089 | 0.125 | 0.012 | 7.74E-01 |
| FCA441^a^ | 0.619 | 0.680 | **0.007** | 1.65E-01 |
| FCA453 | 0.727 | 0.743 | 0.167 | 1.13E-01 |
| F124 | 0.636 | 0.844 | **0.000** | 4.49E-02 |
| F98 | 0.241 | 0.296 | **0.009** | 5.17E-01 |
| *Mean* | *0.575* | *0.689* |  |  |

**Table S2** Sets of ten WorldClim bioclimatic variables selected (Pearson's correlation coefficient r < 0.85) to build ecological niche models for each genetic group: CN (cluster “North”) and CS (cluster “South”). Note that six variables are present for both clusters (Bio01, Bio02, Bio03, Bio09, Bio12 and Bio14). Only seven of the ten variables were retained in the best supported ecological niche model for each genetic cluster.

| CN | CS |
| --- | --- |
| Bio01 = Annual Mean Temperature | Bio01 = Annual Mean Temperature |
| Bio02 = Mean Diurnal Range | Bio02 = Mean Diurnal Range |
| Bio03 = Isothermality | Bio03 = Isothermality |
| Bio04 = Temperature Seasonality | Bio06 = Min Temperature of Coldest Month |
| Bio05 = Max Temperature of Warmest Month | Bio09 = Mean Temperature of Driest Quarter |
| Bio08 = Mean Temperature of Wettest Quarter | Bio12 = Annual Precipitation |
| Bio09 = Mean Temperature of Driest Quarter | Bio14 = Precipitation of Driest Month |
| Bio12 = Annual Precipitation | Bio15 = Precipitation Seasonality |
| Bio13 = Precipitation of Wettest Month | Bio18 = Precipitation of Warmest Quarter |
| Bio14 = Precipitation of Driest Month | Bio19 = Precipitation of Coldest Quarter |


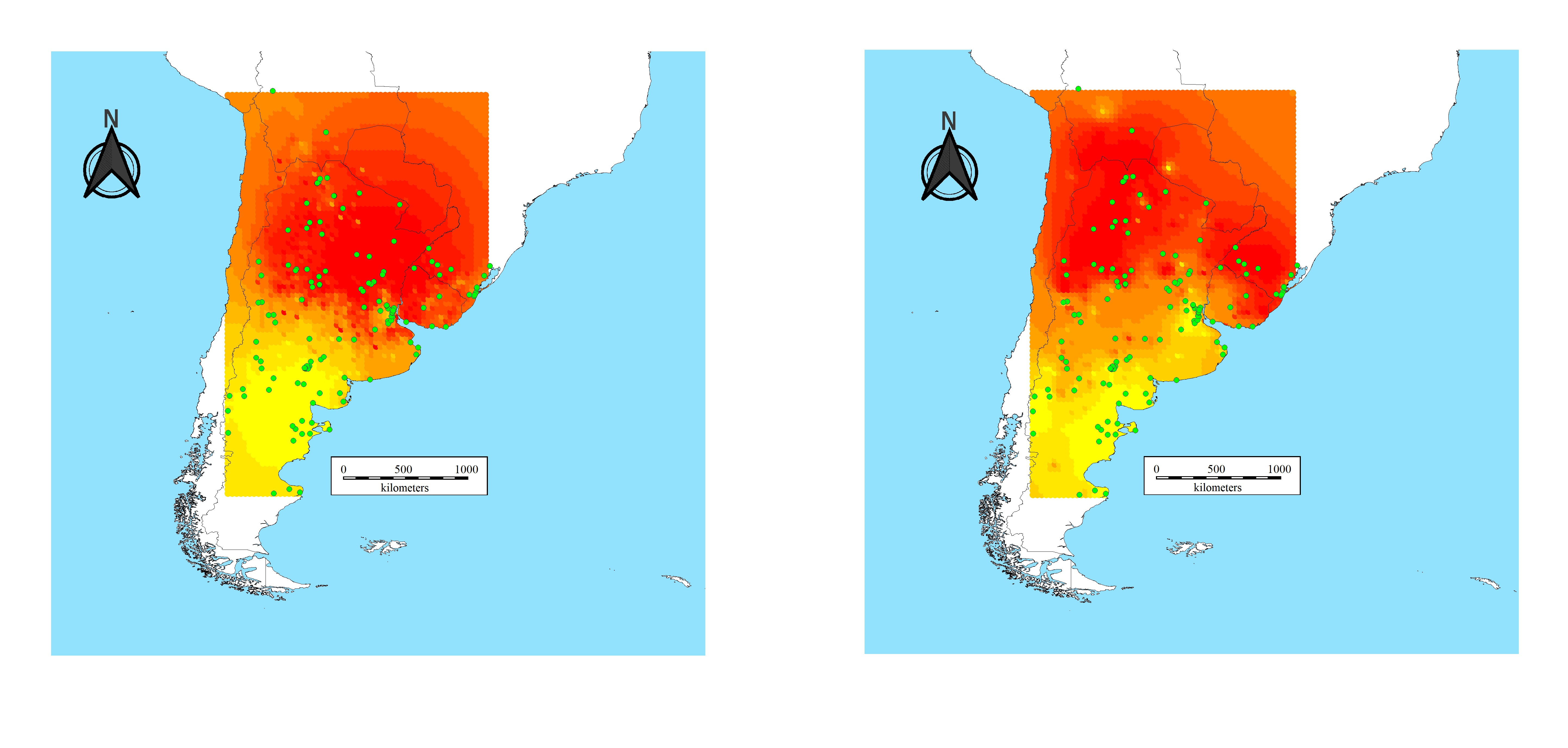


**Figure S1** Results of the Landscape Shape Interpolation analysis visualized using DIVA-GIS v.7.5.0 (http://www.diva-gis.org/) for (a) pairwise connectivity network and (b) Delaunay connectivity network. In yellow: lower genetic difference between individuals. In red: higher genetic difference between individuals. Circles: collection site of the samples.


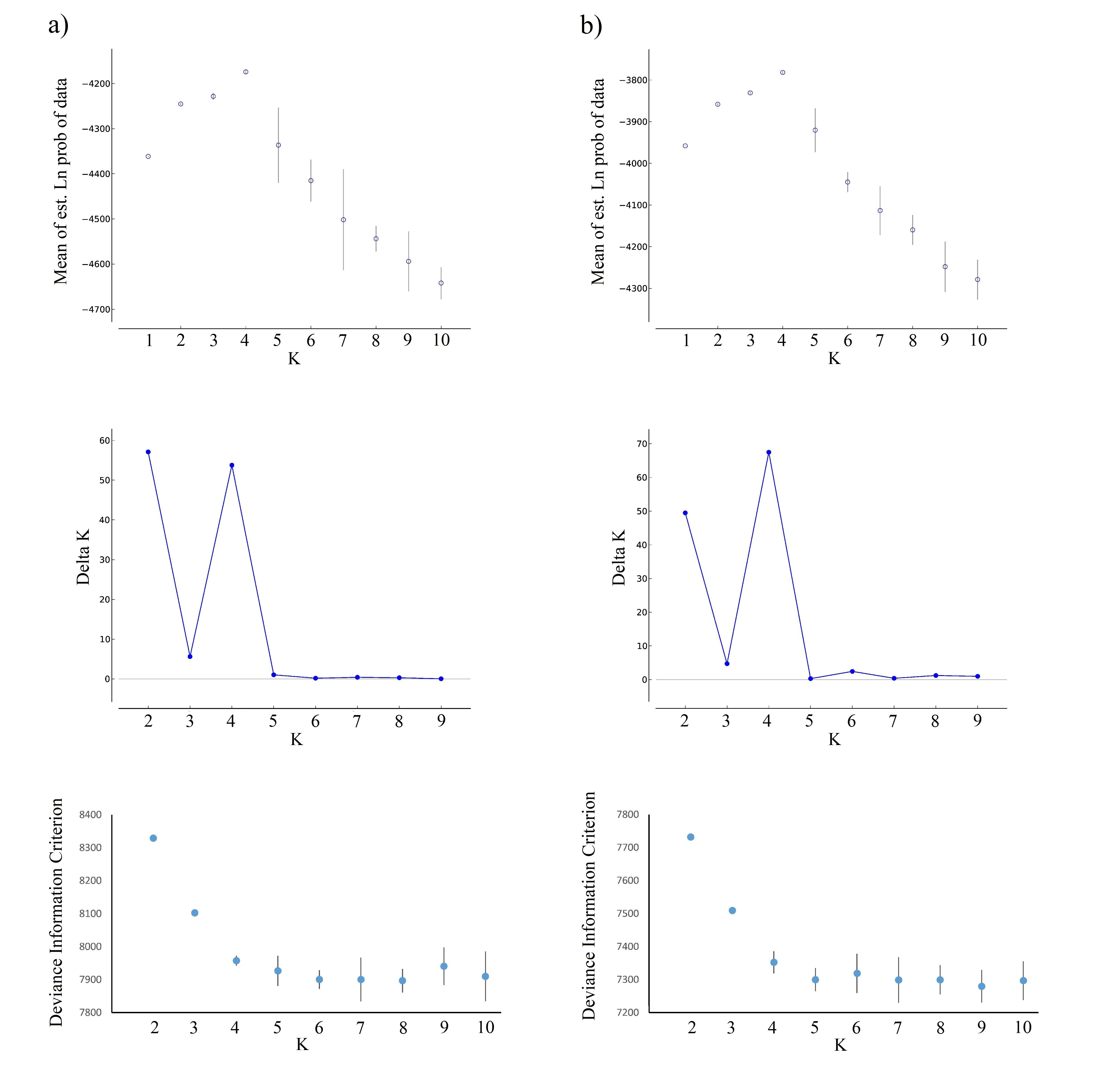


**Figure S2** Results of the Bayesian clustering programs to assist in the selection of the genetic structure scenario, where K represents the number of clusters: (a) using all the samples and (b) only samples collected in or after 1990. Top: mean log probability (STRUCTURE runs); center: Evanno’s ΔK (STRUCTURE runs); bottom: deviance information criterion (TESS runs).

**Figure S3** Response curves for the bioclimatic variables not shared between the best ecological niche model for genetic cluster “North” (a) and the one for cluster “South” (b). The vertical axis shows predicted value of suitability for Geoffroy’s cat (*Leopardus geoffroyi*).


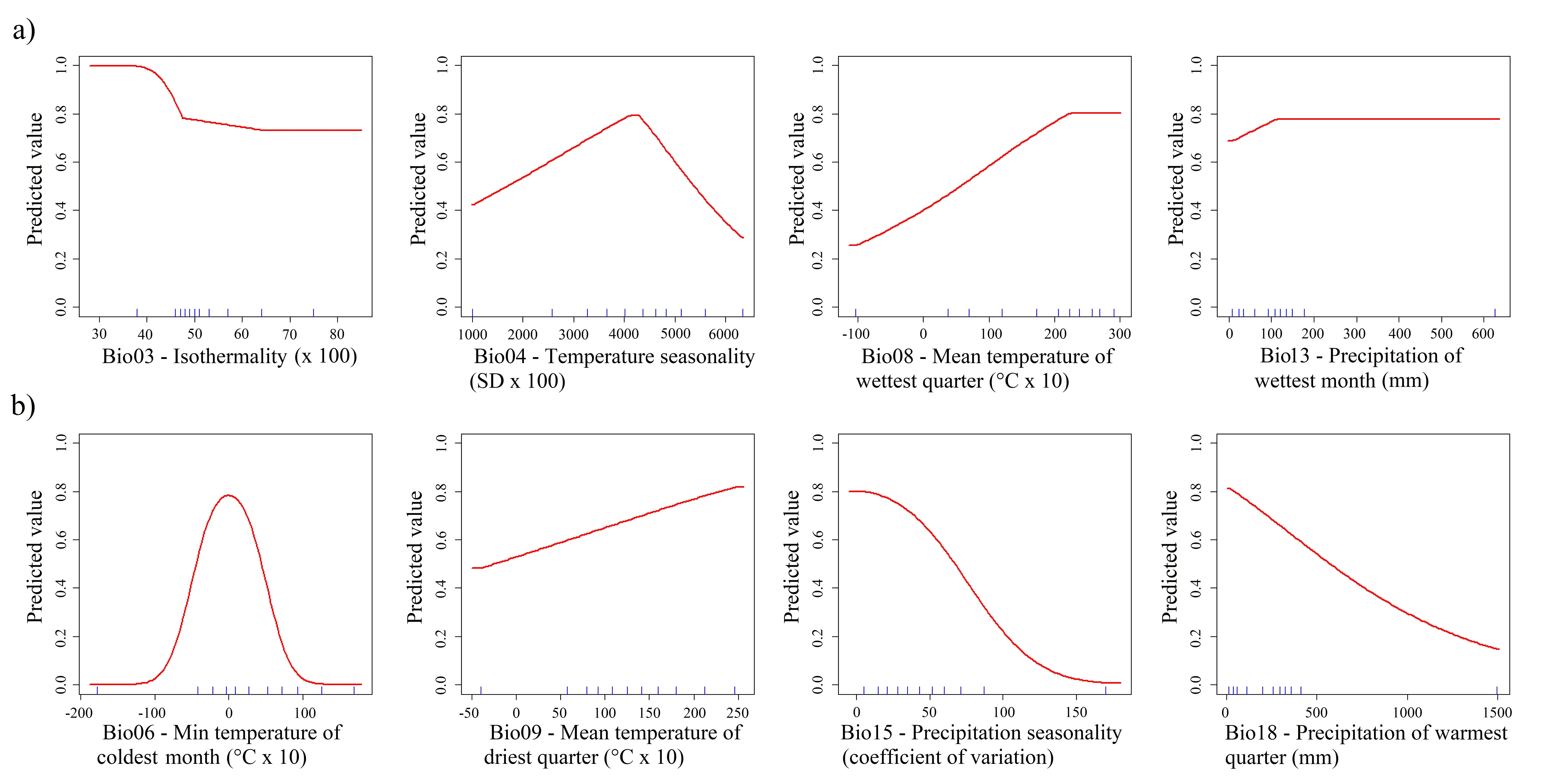


**Figure S4** Response curves for the three bioclimatic variables shared between the best ecological niche model for genetic cluster “North” (CN) and the one for cluster “South” (CS). The vertical axis shows predicted value of suitability for Geoffroy’s cat (*Leopardus geoffroyi*).


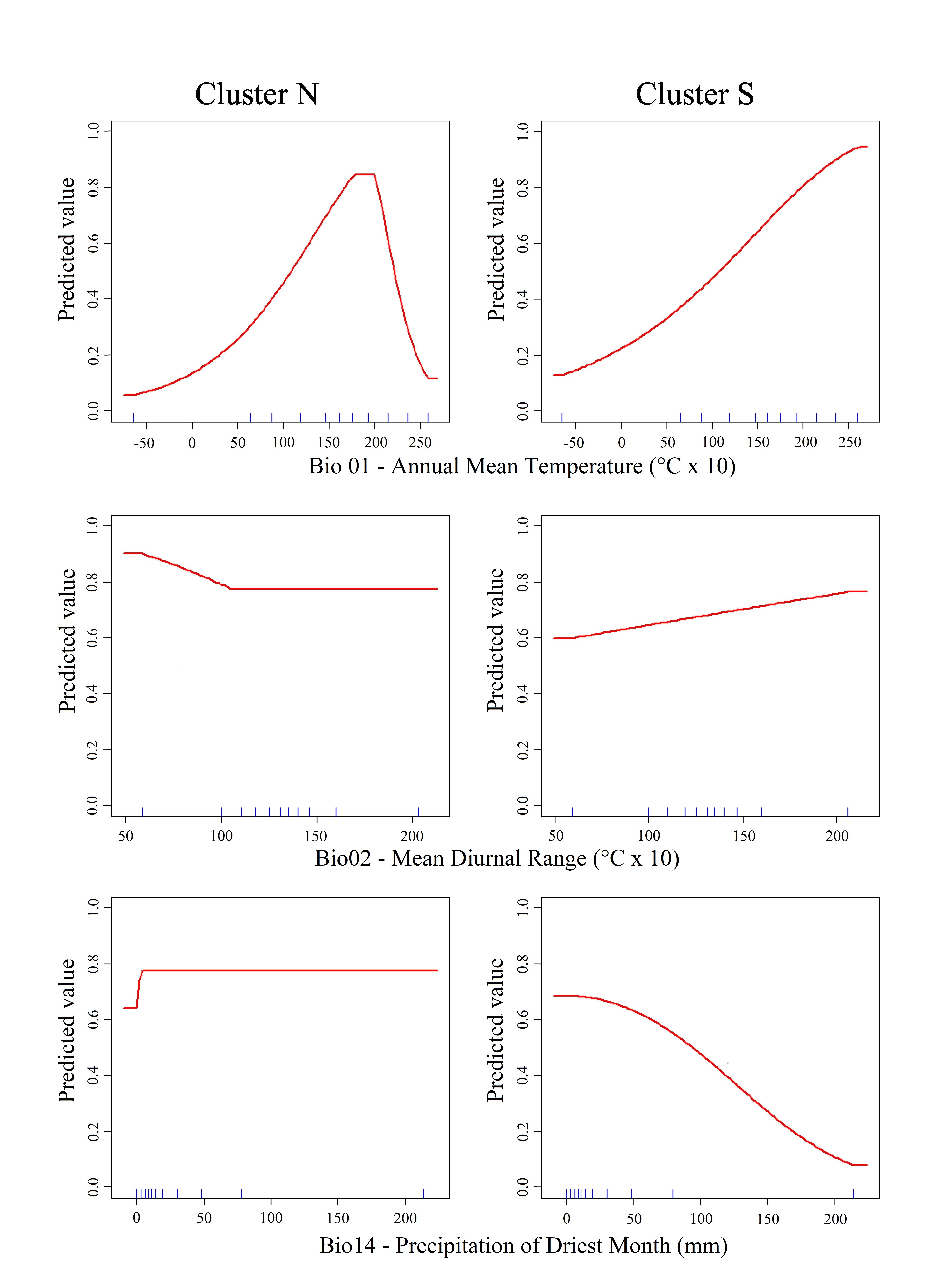

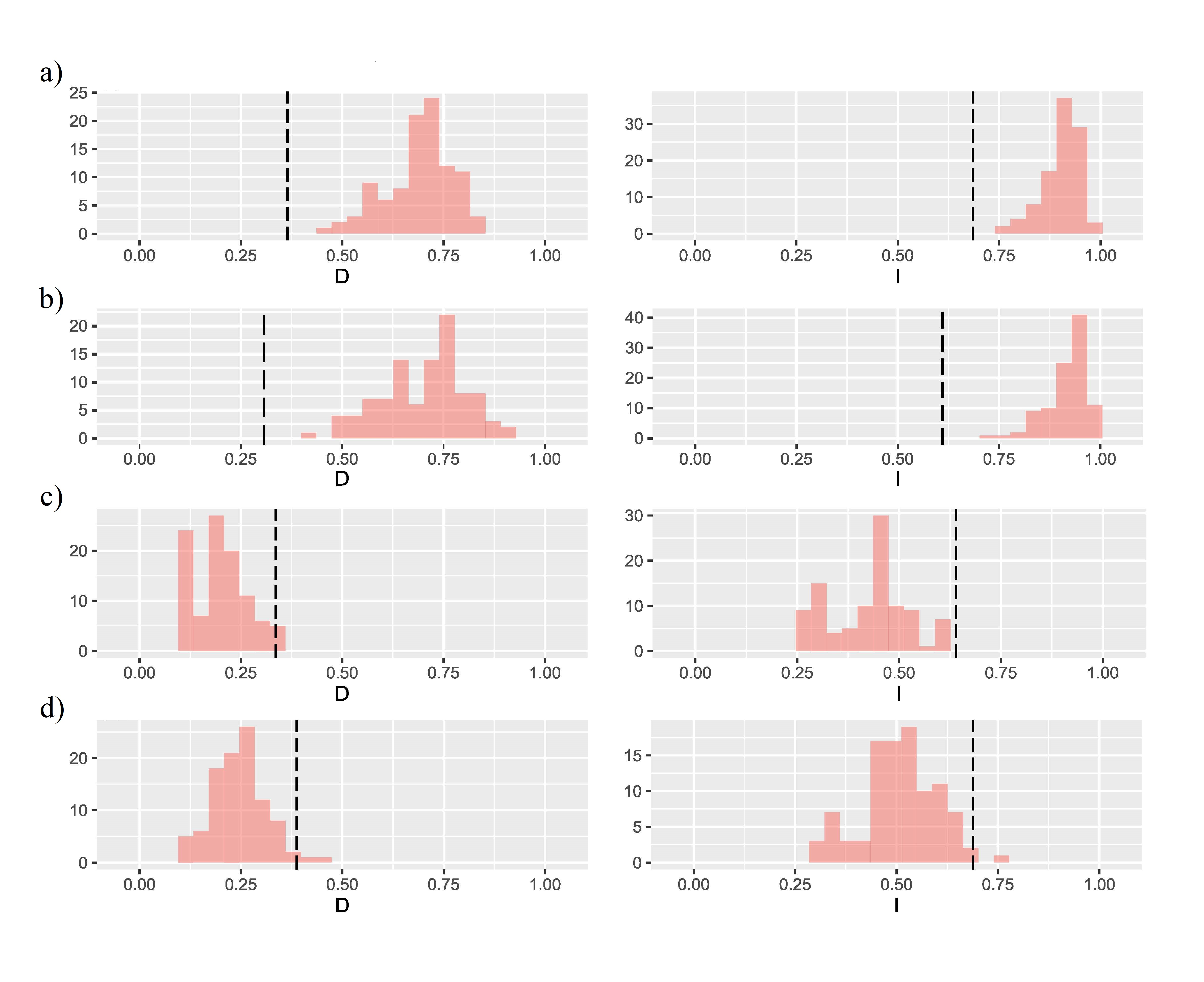


**Figure S5** Statistical comparisons between ecological niche models for both genetic clusters, using Schoener’s D (left) and Warren’s I (right) statistics. The dashed line indicates the empirical measure for each test statistic and the pink histogram shows the null distribution. Results are considered significant if the empirical measure falls outside 95% of the null distribution. (a) Empirical values for the identity test; D and I test statistics were significantly different than the null distribution (p-value=0.01), indicating niche differentiation between clusters. (b) Empirical values for the symmetrical background test; D and I test statistics were significantly different than the null distribution (p-value<0.01), indicating that the distribution of individuals in each group is not random with respect to the available climate characteristics. (c) Lineal and (d) Blob range-break test results for cluster “north” (CN) versus cluster “south” (CS). Empirical values for the D and I test statistics were significantly different than the null distribution (p-value<0.05), indicating that the climate characteristics differ between these two geographic areas.
